# Are Northern Hemisphere boreal forest fires more sensitive to future aerosol mitigation than to greenhouse gas–driven warming?

**DOI:** 10.1126/sciadv.adl4007

**Published:** 2024-03-29

**Authors:** Robert J. Allen, Bjørn H. Samset, Laura J. Wilcox, Rosie A. Fisher

**Affiliations:** ^1^ Department of Earth and Planetary Sciences, University of California Riverside, Riverside, CA 92521 USA.; ^2^ CICERO Center for International Climate and Environmental Research in Oslo, Oslo, Norway.; ^3^ National Centre for Atmospheric Science, University of Reading, Reading, UK.

## Abstract

Considerable interest exists in understanding how climate change affects wildfire activity. Here, we use the Community Earth System Model version 2 to show that future anthropogenic aerosol mitigation yields larger increases in fire activity in the Northern Hemisphere boreal forests, relative to a base simulation that lacks climate policy and has large increases in greenhouse gases. The enhanced fire response is related to a deeper layer of summertime soil drying, consistent with increased downwelling surface shortwave radiation and enhanced surface evapotranspiration. In contrast, soil column drying is muted under increasing greenhouse gases due to plant physiological responses to increased carbon dioxide and by enhanced melting of soil ice at a depth that increases soil liquid water. Although considerable uncertainty remains in the representation of fire processes in models, our results suggest that boreal forest fires may be more sensitive to future aerosol mitigation than to greenhouse gas–driven warming.

## Teaser

Boreal forest fires may be more sensitive to future aerosol mitigation than to continued increases in greenhouse gases.

## INTRODUCTION

Fire is a fundamental component of Earth system, altering ecosystems and affecting air quality and atmospheric composition (*1*–*3*). Continued climate change, including intensified drought and more frequent heatwaves, is expected to enhance fire weather and increase wildfire activity in the coming years (*4*–*6*). For example, in the Northern Hemisphere (NH) boreal forests, climate warming, and drying have led to heightened wildfire activity (*7*, *8*), with large increases in the annual area burned in Canada and Alaska over recent decades (*9*, *10*) and under future projections (*11*–*14*). However, the NH boreal regions as a whole (poleward of 60°N) have a nonsignificant increasing trend of ~2.5 % year^−1^, while the boreal North America and boreal Asia regions as a whole have decreasing trends (although this includes many areas that are not forest) (*15*). Wildfire carbon emissions in the boreal forest regions are also strongly driven by fuel availability (*16*).

How individual climate drivers, such as anthropogenic aerosols, affect wildfire activity is not well known. Solar geoengineering experiments including stratospheric aerosol injection resulted in a decrease in wildfires (*17*) due to decreasing surface temperature and wind speed, along with increasing relative humidity (RH) and soil water. Additional analysis has suggested that aerosol-driven cooling throughout the 20th century balanced greenhouse gas (GHG)–driven increases in extreme fire weather conditions (*18*). However, under future aerosol reductions, combined with continued increases in GHGs, extreme fire weather is expected to experience unprecedented increases (*18*). As with some of the above studies (*14*), these latter results are based on the Canadian Forest Fire Weather Index (FWI) (*19*), which quantifies fire weather conditions based on daily maximum temperature, precipitation, RH, and surface wind. FWI, however, does not take account of other drivers of fire activity, such as live and dead fuel loads, fire suppression, or more complex changes in surface biophysics and hydrology.

Here, we use the Community Earth System Model version 2 (CESM2) (*20*) whose land component, the Community Land Model version 5 (CLM5) (*21*), includes an explicit representation of fire activity (Materials and Methods) (*22*–*24*) to quantify the impact of 2015–2060 anthropogenic aerosol mitigation on fire carbon emissions (FIREC). Changes in climate drivers impact fire carbon emissions in our simulations, but fire emissions do not subsequently feedback to impact the climate. Although our approach—as with prior analyses—also contains considerable uncertainties, CESM2 can reasonably reproduce observed fire statistics (*17*, *24*, *25*), including in the NH boreal forest region (Materials and Methods). We find a robust FIREC increase in the NH boreal forest region under aerosol mitigation, which is larger than that under the high-GHG baseline experiment, due to a deeper layer of summertime soil drying associated with increased surface solar radiation and enhanced evapotranspiration.

## RESULTS

### Aerosol mitigation

Aerosol mitigation is quantified as specified under the Regional Aerosol Model Intercomparison Project (RAMIP) (*26*). The baseline simulation (“ssp370”) is driven by anthropogenic emissions (e.g., GHGs, aerosols and precursor gases, and ozone) and land use/land change from the Shared Socioeconomic Pathway 3-7.0 (SSP37.0) (*27*–*29*), which lacks climate policy and has relatively weak levels of air quality control measures. The perturbation experiment (“ssp370-126aer”) is identical (e.g., the same increase in GHGs) but uses anthropogenic aerosol and precursor gas emissions from SSP1-2.6, which features relatively strong levels of air quality control measures. Throughout this manuscript, the term “aerosol mitigation” refers to the difference of these two simulations (i.e., ssp370-126aer-ssp370; Table 1). Because both are identical in all ways except their aerosol/precursor gas emissions, the difference quantifies the climate effects of strong versus weak air quality control, i.e., aerosol mitigation. A similar experimental design was adopted by the Aerosol Chemistry Model Intercomparison Project (*30*), where near-term climate forcer (NTCF) mitigation was quantified as the difference between the ssp370 and ssp370-lowNTCF experiments (*31*, *32*).

**Table 1.** Definition of CESM2 experiments used in this study.

Experiment name	Description	
ssp370	Weak air quality control and low climate mitigation	
ssp370-126aer	Strong air quality control and low climate mitigation	
**Mitigation signal**	**Description**	**Abbreviation**
ssp370-126aer−ssp370	Climate effects of strong versus weak air quality control	Aerosol mitigation

As expected, aerosol mitigation yields significant aerosol optical depth (AOD) decreases over the global land, including the NH extratropics (figs. S1 to S3 and note S1). In contrast, relatively small and nonsignificant NH extratropical AOD changes occur under ssp370. In turn, the AOD decrease under aerosol mitigation is associated with significant warming over the NH boreal forest region (fig. S4), which is about half as large as the corresponding warming (largely due to the increase in GHGs) under ssp370. As aerosols decrease surface solar radiation through both aerosol-radiation interactions (e.g., direct scattering/absorbing of incoming sunlight) and aerosol-cloud interactions (e.g., brighter and longer-lived clouds), aerosol mitigation is associated with increases in surface solar radiation (to be discussed below), which drives the warming.

### Fire responses

**Fig. 1.**
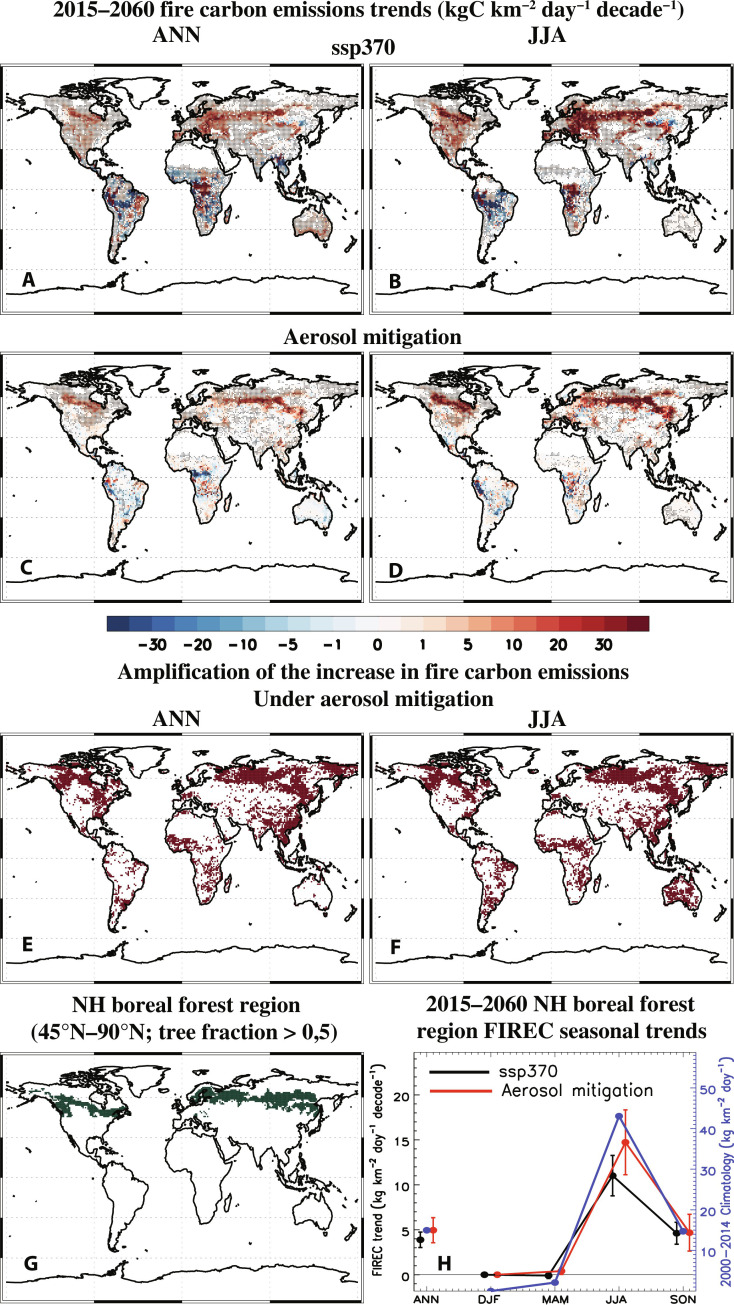
2015–2060 fire carbon emissions trend maps. (**A** and **C**) Annual mean and (**B** and **D**) June-July-August (JJA) mean fire carbon emissions trends (kgC km^−2^ day^−1^ decade^−1^) for (A and B) ssp370 and (C and D) aerosol mitigation (i.e., ssp370-126aer-ssp370). Dots represent a significant response at the 90% confidence level based on a standard two-tailed *t* test. Also included are (**E**) annual and (**F**) JJA maps showing amplification of the increase in fire carbon emissions under aerosol mitigation (relative to ssp370). Red shading denotes regions where aerosol mitigation yields larger FIREC increases relative to ssp370 (i.e., percent change exceeds 100%) or aerosol mitigation yields positive FIREC trends, whereas ssp370 yields negative trends. (**G**) NH boreal forest region (i.e., dark green shading) as defined here, as grid boxes over land from 45–90°N with at least 50% tree fraction. (**H**) 2015–2060 NH boreal forest region FIREC seasonal trends for both ssp370 (black) and aerosol mitigation (red). The 2000–2014 climatology is also included (blue).

Figure 1 shows annual (ANN) and June-July-August (JJA) mean model mean (averaged over the 10 ensemble members) FIREC trend maps for ssp370 and aerosol mitigation. Globally, the ANN FIREC trends are not significant for both ssp370 and aerosol mitigation at −0.11 ± 0.29 and 0.36 ± 0.41 kgC km^−2^ day^−1^ decade^−1^, respectively. The tropics (30°S to 30°N) show significant negative FIREC trends at −2.5 ± 0.58 kgC km^−2^ day^−1^ decade^−1^ for ssp370 and nonsignificant negative FIREC trends at −0.58 ± 0.78 kgC km^−2^ day^−1^ decade^−1^ under aerosol mitigation. In contrast, the NH mid- and high-latitudes (30°N to 90°N) yield significant positive trends for both ssp370 and aerosol mitigation at 2.3 ± 0.2 and 1.5 ± 0.3 kgC km^−2^ day^−1^ decade^−1^, respectively. Figure 1 shows that much of this 30°N to 90°N increase in FIREC occurs in the NH boreal forest region (Fig. 1G), which we now focus on.

In the NH boreal forest region, FIREC trends peak during NH summertime (JJA; Fig. 1H), and interestingly, aerosol mitigation yields larger (but not significantly so) FIREC increases than does ssp370. The boreal forest region ANN FIREC trend is 3.89 ± 0.86 kgC km^−2^ day^−1^ decade^−1^ under ssp370 versus 4.95 ± 1.39 kgC km^−2^ day^−1^ decade^−1^ under aerosol mitigation. The corresponding ANN percent changes are 119.4% for ssp370 and 152.2% for aerosol mitigation. Percent changes are estimated relative to the 2000–2014 climatology, which is calculated from the CESM2 Large Ensemble historical simulations (*33*). The corresponding ANN FIREC climatology for the NH boreal forest region is 15.0 kgC km^−2^ day^−1^. We note that this represents about 40% of the global FIREC climatology of 39.6 kgC km^−2^ day^−1^. During JJA, the corresponding trends increase to 11.0 ± 2.2 and 14.7 ± 3.6 kgC km^−2^ day^−1^ decade^−1^ (117.7% and 157.4%). The FIREC increase is also robust across realizations, as all 10 ensemble members (for both ssp370 and aerosol mitigation) yield an increase in boreal forest region ANN and JJA fire carbon emissions (fig. S5 shows the percentage of realizations that yield a positive trend under aerosol mitigation for ANN and JJA). Normalizing the boreal forest region FIREC trends by the corresponding warming trends yields significantly larger values under aerosol mitigation as opposed to ssp370 at 15.0 ± 4.5 versus 7.0 ± 1.6 kgC km^−2^ day^−1^ K^−1^ for ANN and 38.9 ± 9.0 versus 17.2 ± 3.8 kgC km^−2^ day^−1^ K^−1^ for JJA, respectively. Similar results exist for fire burned area (fig. S6).

Figure 1 (E and F) shows regions where aerosol mitigation yields amplified (Materials and Methods) FIREC trends. Amplification of the increase in fire carbon emissions under aerosol mitigation (relative to ssp370) occurs when aerosol mitigation yields larger FIREC increases relative to ssp370 (i.e., percent change exceeds 100%) or aerosol mitigation yields positive FIREC trends, whereas ssp370 yields negative trends. Much of boreal forest region including parts of Canada and Russia features red colors, as does eastern/northeastern China. Aerosol mitigation yields ANN FIREC increases more than 81.6% of the boreal forest region, with FIREC amplification (relative to ssp370) more than 51.4% of the region. In comparison, ssp370 yields FIREC amplification (relative to aerosol mitigation) more than 39.9% of the boreal forest region.

To confirm the ssp370 signal is largely related to the increase in GHGs (as opposed to changes in aerosols), we also briefly analyze the CESM2 single forcing experiments (*34*), which are likewise driven by SSP3-7.0 emissions (in both GHG-only and aerosol-only experiments). On the basis of 15 ensemble members from 2015–2050 (when these simulations end), GHGs (based on SSP3-7.0) alone yield a boreal forest region FIREC increase of 3.70 ± 1.30 kgC km^−2^ day^−1^ decade^−1^, which is nearly the same as that discussed above based on ssp370. Furthermore, the corresponding FIREC trends under industrial aerosol emissions and biomass burning aerosol emissions (based on SSP3-7.0) are both much smaller and not significant at 0.08 ± 0.68 and 0.46 ± 1.2 kgC km^−2^ day^−1^ decade^−1^. Thus, aerosol contributions to FIREC changes under ssp370 are negligible, and we therefore interpret the ssp370 FIREC changes as a GHG response.

### Mechanisms of enhanced wildfire activity

**Fig. 2.**
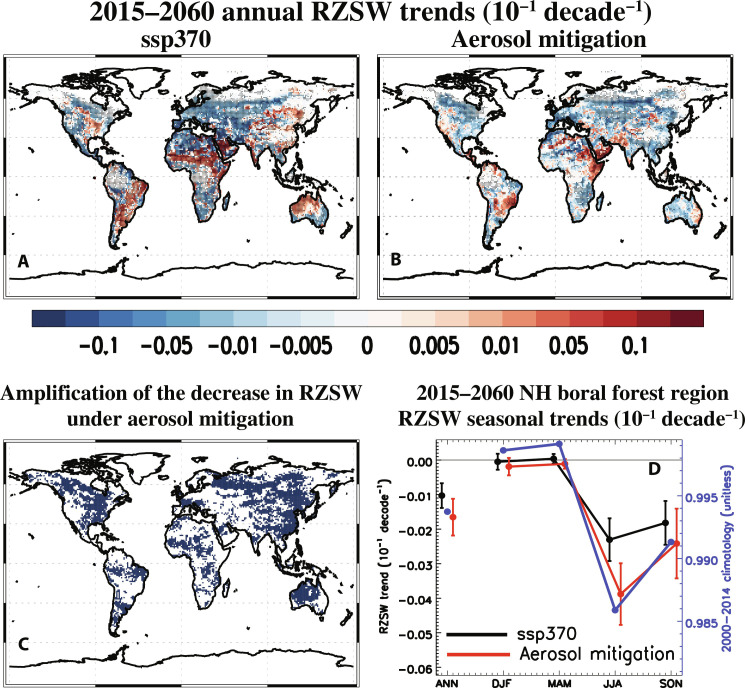
2015–2060 RZSW trend maps. Annual mean RZSW trends (10^−1^ decade^−1^) for (**A**) ssp370 and (**B**) aerosol mitigation (i.e., ssp370-126aer-ssp370). Dots represent a significant response at the 90% confidence level based on a standard two-tailed *t* test. Also included (**C**) is the corresponding map showing amplification of the decrease in RZSW under aerosol mitigation (relative to ssp370). Blue shading denotes regions where aerosol mitigation yields larger RZSW decreases relative to ssp370 (i.e., percent change exceeds 100%) or aerosol mitigation yields negative RZSW trends, whereas ssp370 yields positive trends. (**D**) 2015–2060 NH boreal forest region RZSW seasonal trends for both ssp370 (black) and aerosol mitigation (red). The 2000–2014 climatology is also included (blue).

Several factors are important for wildfire activity, including soil moisture, surface RH, precipitation, air temperature, solar radiation and windspeed, as well as vegetation and fuel characteristics (Materials and Methods). Root-zone soil wetness (RZSW)—a key driver of fire behavior, which depends on the soil water potential of each soil layer, the root distribution of the plant functional type, and a plant dependent response to soil water stress (*35*)—features significant negative trends under aerosol mitigation over many areas, most notably the boreal forest region (Fig. 2), and the pattern of decreasing RZSW closely corresponds to the areas of FIREC increases (Fig. 1). Over the NH boreal forest region, ANN RZSW trends are −0.017 ± 0.005 versus −0.010 ± 0.004 10^−1^ decade^−1^ under aerosol mitigation and ssp370, respectively, and these increase (in magnitude) during JJA (Fig. 2D). Furthermore, aerosol mitigation yields amplified (negative) RZSW trends relative to ssp370 throughout the boreal forest region (Fig. 2C), with RZSW amplification over 59.5% of the region (ssp370 yields RZSW amplification, relative to aerosol mitigation, more than 32.4% of the boreal forest region). Soil water in the upper 10 cm [SW10CM; used by some prior studies (*17*, *25*)] yields broadly similar conclusions as RZSW, but the SW10CM trend amplification under aerosol mitigation is weaker (fig. S7C), and there is also less spatial correspondence between SW10CM decreases (fig. S8, B and G) and FIREC increases (Fig. 1). Aerosol mitigation yields relatively weak decreases in near-surface RH, with minimal regions of negative trend amplification relative to ssp370. Over the boreal forest region, annual mean RH decreases by −0.09 ± 0.05% decade^−1^ under aerosol mitigation compared to −0.28 ± 0.04% decade^−1^ under ssp370 (fig. S7E; similar conclusions exist for JJA). Boreal forest region near-surface warming is also weaker under aerosol mitigation (fig. S7D). For both ssp370 and aerosol mitigation, surface wind speed decreases during ANN and JJA (fig. S7F). In terms of precipitation, aerosol mitigation yields increased precipitation for the boreal forest region in all seasons, with the largest (but not significantly so) increase during JJA, whereas ssp370 features a decrease in JJA precipitation (ssp370 precipitation increases in the other three seasons; fig. S7G). Thus, seasonal mean and annual mean changes in these other fire-related climate parameters do not change in way that would favor enhanced fire activity (relative to ssp370) under aerosol mitigation. However, changes in extreme values of hydrologically relevant parameters (e.g., subseasonal drought frequency/intensity) may also be important.

**Fig. 3.**
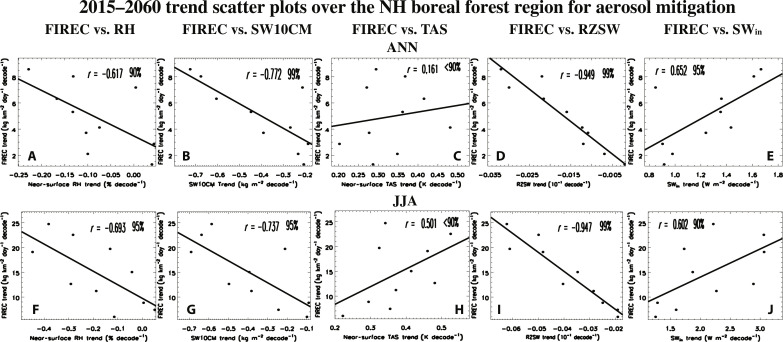
2015–2060 aerosol mitigation trend scatterplots. 2015–2060 boreal forest region fire carbon emissions (FIREC) trends (kgC km^−2^ day^−1^ decade^−1^) versus corresponding trends in (**A** and **F**) near-surface RH (% decade^−1^), (**B** and **G**) soil water upper 10 cm [SW10CM; (kg m^−2^ decade^−1^)], (**C** and **H**) near-surface air temperature (TAS; [K decade^−1^]); (**D** and** I**) RZSW (10^−1^ decade^−1^), and (**E** and **J**) surface downwelling shortwave radiation (SW_in_) for the (A to E) annual mean and (F to J) JJA mean. Each dot represents the trend from one of the 10 ensemble members under aerosol mitigation (i.e., ssp370-126aer-ssp370). Also included is the correlation coefficient, *r*, and its significance based on a standard *t* test.

To further evaluate the role of different factors, Figure 3 shows 2015–2060 boreal forest region aerosol mitigation (i.e., ssp370-126aer minus ssp370) trend scatterplots, where the FIREC trends (from each of the 10 ensemble members) are compared to the corresponding trends of several climate variables (more generally, fig. S9 shows the corresponding spatial maps). A significant correlation implies the importance of the climate variable to FIREC trends. Significant negative correlations exist for both the ANN and JJA mean between FIREC trends and near-surface RH (Fig. 3, A and F), SW10CM (Fig. 3, B and G) and, particularly, RZSW (Fig. 3, D and I). For the latter two relationships, the ANN correlations are −0.77 and − 0.95; for JJA, the corresponding correlations are similar at −0.74 and −0.95. Positive correlations exist between FIREC and near-surface air temperature trends (TAS; Fig. 3, C and H), but they are weak, especially for ANN at 0.16. Similar correlations generally occur under ssp370 (fig. S10), where the ANN FIREC and RZSW trend correlation is −0.96; the corresponding FIREC versus near-surface RH trend correlation is −0.80. We also note a significant positive correlation between FIREC trends and surface downwelling shortwave radiation (SW_in_) trends under aerosol mitigation (Fig. 3, E and J). The corresponding correlations under ssp370, however, are not significant (fig. S10, E and J). The importance of SW_in_ under aerosol mitigation is expanded upon below.

The role of other factors, including human ignition/suppression and changes in vegetation indices, is minimal for the NH boreal forest region (note S2). For example, the change in vegetation indices (e.g., leaf area index)—despite increasing under both ssp370 and aerosol mitigation—do not significantly correlate with FIREC trends. This is consistent with (*25*), where CO_2_ biogeochemical effects (e.g., the CO_2_ fertilization effect on vegetation) result in an increase in vegetation indices (e.g., net primary productivity) in the NH boreal forest region in CESM2, but a negligible change in FIREC occurs. This is, however, model dependent as most other models yield FIREC increases [see also (*36*)].

Thus, changes in RZSW appear to be the most important driver of FIREC trends under aerosol mitigation (and ssp370). More importantly, these analyses suggest the dominant reason for FIREC amplification under aerosol mitigation is due to RZSW. This is supported by the strong negative correlations between FIREC and RZSW trends in the boreal forest region, and the significantly larger decreases in RZSW under aerosol mitigation (Fig. 2). Despite only 10 realizations in the correlation analysis, we note that the RZSW versus FIREC trend points fall very close to a linear line and yield a correlation of −0.95 (Fig. 3, D and I).

### Mechanisms of soil column drying

The relatively large decreases in RZSW under aerosol mitigation (in particular, despite smaller warming compared to ssp370) is related to increased SW_in_ (fig. S8, C and H), and subsequent increases in surface latent heat flux (fig. S8, D and I) associated with evapotranspiration, and in particular canopy transpiration (fig. S8, E and J) over most world regions. In the context of evapotranspiration, the boreal forest region is an energy limited as opposed to soil moisture limited region (*37*, *38*), i.e., evapotranspiration is largely controlled by energy availability (e.g., SW_in_). This is illustrated in fig. S11 (A and B), which shows aerosol mitigation correlation maps based on the 2015–2060 JJA model mean (average over the 10 ensemble members) time series between SW_in_ and both latent heat flux and canopy transpiration. Significant positive correlations exist between JJA SW_in_ and both latent heat flux and canopy transpiration in the boreal forest region. These correlations are also larger than those associated with near-surface air temperature and latent heat flux/canopy transpiration (fig. S11, C and D). Thus, latent heat flux and canopy transpiration variations are likely largely controlled by variations in SW_in_ in the boreal forest region.

**Fig. 4.**
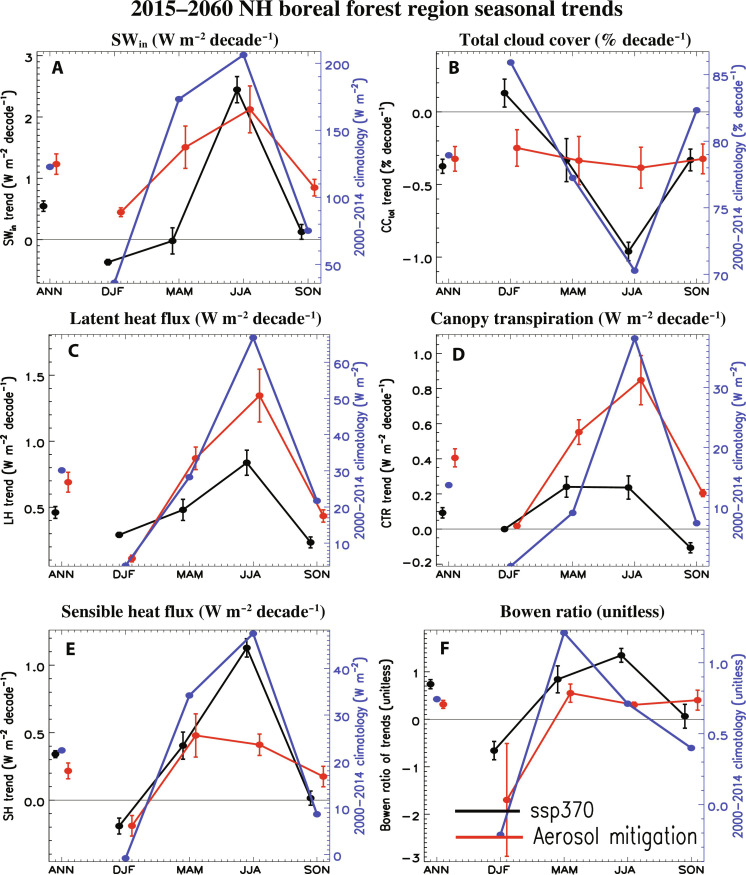
2015–2060 NH boreal forest region seasonal trends. NH boreal forest region seasonal trends for (**A**) surface downwelling shortwave radiation [SW_in_; (W m^−2^ decade^−1^)], (**B**) total cloud cover (% decade^−1^), (**C**) surface latent heat flux (W m^−2^ decade^−1^), (**D**) canopy transpiration (W m^−2^ decade^−1^), (**E**) surface sensible heat flux (W m^−2^ decade^−1^), and (**F**) Bowen ratio [ratio of sensible heat flux trend to latent heat flux trend (unitless)]. In addition to 2015–2060 ssp370 (black) and aerosol mitigation (i.e., ssp370-126aer-ssp370; red) trends, the 2000–2014 climatology (blue) is also included. Seasons include December-January-February (DJF), March-April-May (MAM), JJA, and September-October-November (SON). Also included is the annual mean (ANN). Error bars show the 90% confidence interval of the trend, estimated as $\frac{1.65\times \sigma }{\sqrt{n-1}}$ , where σ is the SD across the trends and *n* is the number of trends (i.e., 10).

As mentioned above, aerosol mitigation yields large increases in SW_in_ (Fig. 4A). During JJA, however, both aerosol mitigation and ssp370 yield similar increases at 2.1 ± 0.38 and 2.4 ± 0.21 W m^−2^ decade^−1^, respectively. The ssp370 SW_in_ increase is related to changes in cloud cover, with total cloud cover decreasing by −0.96 ± 0.06% decade^−1^ (Fig. 4B). The SW_in_ increase under aerosol mitigation is consistent with the large decrease in aerosol emissions and AOD (e.g., fig. S1), as well as aerosol indirect effects on clouds (e.g., total JJA cloud cover decreases by −0.38 ± 0.14% decade^−1^).

There are also large increases in latent heat flux (Fig. 4C) and canopy transpiration (Fig. 4D), which are significantly larger under aerosol mitigation. During JJA, aerosol mitigation yields latent heat flux and canopy transpiration trends of 1.35 ± 0.20 W m^−2^ decade^−1^ and 0.85 ± 0.14 W m^−2^ decade^−1^, respectively. In contrast, ssp370 yields corresponding trends of 0.84 ± 0.10 and 0.24 ± 0.07 W m^−2^ decade^−1^. Most of the latent heat flux increase under aerosol mitigation is related to canopy transpiration increases, as the other components, including canopy and ground evaporation, experience smaller increases (fig. S12, C and D). Thus, aerosol mitigation drives enhanced SW_in_ and latent heat flux, the latter being dominated by increases in canopy transpiration-these increases act to dry the soil.

We note that plant photosynthesis and terrestrial ecosystem productivity is generally more sensitive to diffuse shortwave radiation, as opposed to its direct component (e.g., by increasing the available radiation at the bottom and shaded parts of the canopy) (*39*, *40*). Over the NH boreal forest region, aerosol mitigation yields significant increases in the total (visible and near-infrared) diffuse surface shortwave radiation for all seasons (fig. S13). In contrast, ssp370 yields significant decreases. The diffuse surface shortwave radiation trends are about a factor of 10 smaller than those associated with total surface shortwave radiation. For example, the JJA NH boreal forest region aerosol mitigation diffuse surface shortwave radiation trend is about 0.2 W m^−2^ decade^−1^ relative to the total surface shortwave radiation trend of about 2 W m^−2^ decade^−1^ (e.g., Fig. 4A). This implies the bulk of the increase in surface solar radiation under aerosol mitigation in the NH boreal forest region is due to increases in the direct component, as opposed to the diffuse component. A similar statement also applies to the percent change. For example, the JJA NH boreal forest region aerosol mitigation diffuse surface shortwave radiation change over the 46 years is about 12% of its climatology, whereas the corresponding total surface shortwave radiation change over the 46 years is about 47% of its climatology. However, the JJA percent change in ssp370 diffuse radiation at −41% is nearly as large as the percent change in total surface shortwave radiation at −54%. To the extent that canopy transpiration is more sensitive to diffuse shortwave radiation (as opposed to direct radiation), these results are consistent with the larger increases in canopy transpiration under aerosol mitigation, as opposed to ssp370.

The weaker latent heat flux and canopy transpiration increase under ssp370 is consistent with plant physiological responses to CO_2_, i.e., the CO_2_ fertilization effect (*41*–*44*). This response includes increases in vegetation indices (e.g., leaf area index) but reduced stomatal conductance and plant water use (*45*). In particular, the biogeophysical effects of CO_2_ alone (in the absence of direct radiative warming) have been associated with a vertical redistribution of water (*25*), including more in the soil and less in the atmosphere. This is consistent with ssp370 (i.e., muted increase in latent heat flux and canopy transpiration relative to aerosol mitigation), as large decreases (i.e., ∼30%) in stomatal conductance occur under ssp370 (note S3 and fig. S12, G and H), but not under aerosol mitigation.

The other components of the surface energy balance in the boreal forest region also increase during JJA, but more so under ssp370. This includes the JJA surface sensible heat flux (Fig. 4E), where the trend is 0.41 ± 0.08 W m^−2^ decade^−1^ under aerosol mitigation, with significantly larger increases at 1.12 ± 0.07 W m^−2^ decade^−1^ under ssp370. Aerosol mitigation therefore features significantly larger increases in latent heat flux (Fig. 4C) but significantly smaller increases in sensible heat flux, as compared to ssp370. This leads to a smaller Bowen ratio (ratio of sensible to latent heat flux trend) under aerosol mitigation, at 0.30 ± 0.05 relative to 1.33 ± 0.15 under ssp370 (Fig. 4F). Thus, under aerosol mitigation, a greater proportion of the available energy at the surface (largely SW_in_) goes into evapotranspiration as opposed to sensible heating. This promotes the enhanced soil drying under aerosol mitigation.

**Fig. 5.**
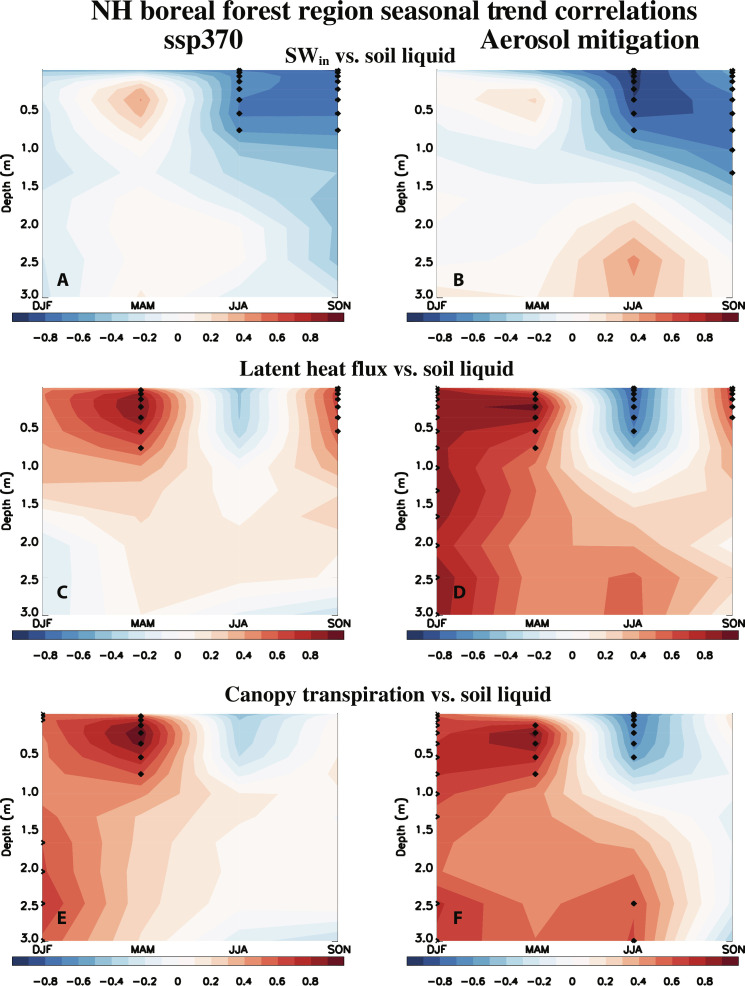
2015–2060 NH boreal forest region season versus soil depth trend correlations. NH boreal forest region depth versus season soil liquid water trend versus (**A** and **B**) SW_in_, (**C** and **D**) latent heat flux, and (**E** and **F**) canopy transpiration trends for (A, C, and E) ssp370 and (B, D, and F) aerosol mitigation (i.e., ssp370-126aer-ssp370). Seasons include DJF, MAM, JJA, and SON. Dots represent a significant correlation at the 90% confidence level based on a standard *t* test.

Trend correlations between the above variables (e.g., SW_in_, latent heat flux and canopy transpiration) and soil liquid water from 0.01 to 3.0 m (Fig. 5) support these notions. Significant negative correlations between SW_in_ and soil liquid water (Fig. 5, A and B) exist down to ~1 m in depth during JJA. These correlations are more negative under aerosol mitigation. For example, the correlations from 0.01 to 0.58 m in depth all exceed −0.83 under aerosol mitigation, with an average correlation of −0.91. Under ssp370, the corresponding correlations range from −0.65 to −0.72, with an average correlation of −0.69. Furthermore, negative JJA correlations exist between surface latent heat flux and soil liquid (Fig. 5, C and D) and between canopy transpiration and soil liquid (Fig. 5, E and F). In both cases, the trend correlations are more negative (and significant) for aerosol mitigation, as opposed to ssp370. We note the positive correlations between latent heat flux and soil liquid water during MAM and SON (and between canopy transpiration and soil liquid water during MAM) near the surface suggest the region is water limited during these seasons (i.e., more soil liquid water allows for more to be evaporated and/or transpired) and/or a role for other processes (e.g., the large soil ice melt and increase in soil liquid water during MAM, as discussed below).

In summary, increasing JJA SW_in_ under aerosol mitigation drives increases in latent heat flux and canopy transpiration, which promotes a decrease in summertime soil liquid water down to ~1 m in depth. As the increase in latent heat flux and canopy transpiration are all larger under aerosol mitigation, this helps to explain the larger decrease in RZSW (relative to ssp370). The additional terms for the surface water balance, including precipitation, do not contribute to the enhanced soil drying under aerosol mitigation (fig. S14 and note S4). For example, precipitation increases in all seasons under aerosol mitigation (fig. S14A), which would act to mute any increases in wildfire activity. The relatively large increase in evapotranspiration (fig. S14B), however, offsets much of this precipitation increase in most seasons, including JJA, leading to a negative precipitation minus evapotranspiration trend (fig. S14E) under aerosol mitigation. We note that the corresponding JJA precipitation minus evapotranspiration trend is slightly more negative under ssp370. As discussed next, larger melting of soil ice under ssp370 helps to explain why RZSW decreases less in ssp370 despite larger decreases in JJA precipitation minus evapotranspiration (relative to aerosol mitigation).

**Fig. 6.**
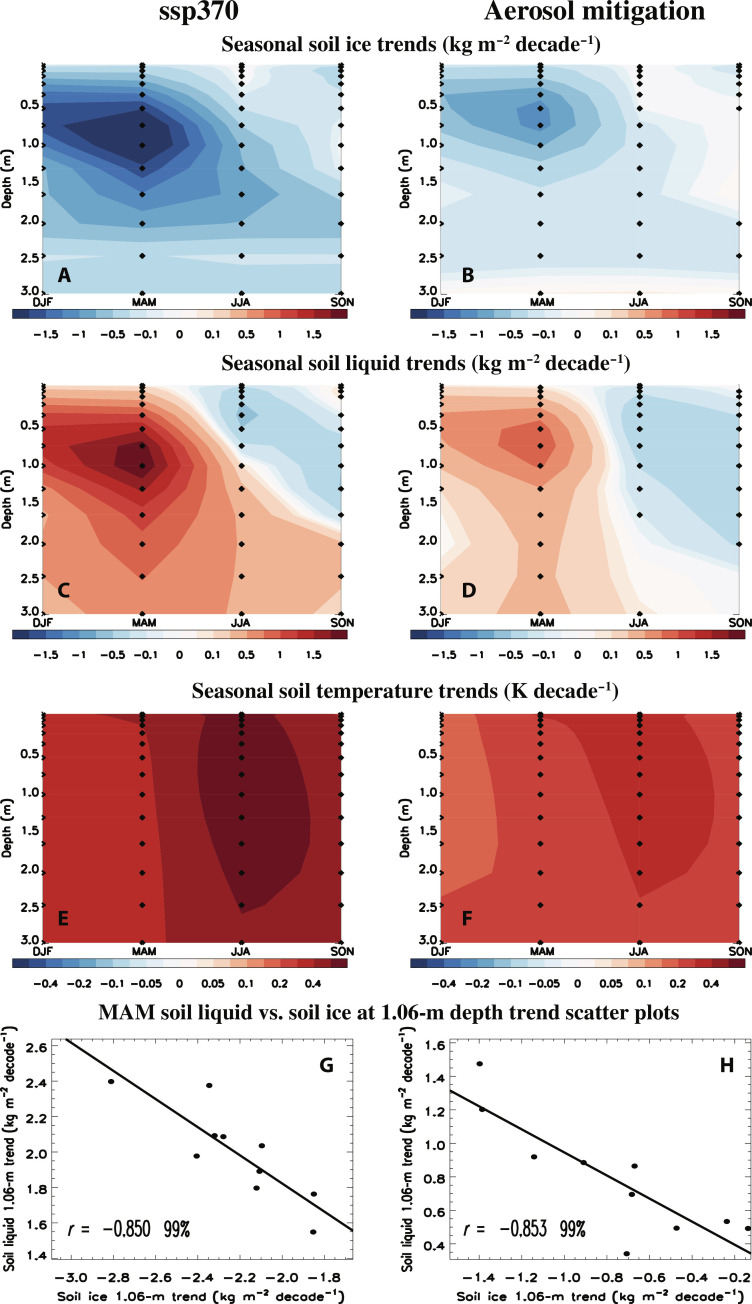
2015–2060 season versus depth soil trend maps. NH boreal forest region season versus depth (**A** and **B**) soil ice (kg m^−2^ decade^−1^), (**C** and **D**) soil liquid water (kg m^−2^ decade^−1^), and (**E** and **F**) soil temperature trends (K decade^−1^) for (A, C, and E) ssp370 and (B, D, and F) aerosol mitigation (i.e., ssp370-126aer-ssp370). Seasons include DJF, MAM, JJA, and SON. Dots represent a significant response at the 90% confidence level based on a standard two-tailed *t* test. Also included are MAM soil liquid water versus soil ice at 1.06-m depth trend scatterplots (across the 10 realizations) for (**G**) ssp370 and (**H**) aerosol mitigation. Also included in (G) and (H) are the correlation coefficient, *r*, and its significance based on a standard *t* test.

An additional reason for the relatively large decrease in RZSW under aerosol mitigation (relative to ssp370) involves soil ice melt. Figure 6 (A and B) shows seasonal trends in the depth of soil ice for the NH boreal forest region. Consistent with warming, both ssp370 and aerosol mitigation yield significant decreases in soil ice in all seasons, with maximum reductions (particularly for ssp370) during NH springtime [March-April-May (MAM)] at ~0.5 to 1 m in depth. The melting leads to an increase in soil liquid water throughout the column (down to 3 m in depth), particularly during MAM (Fig. 6, C and D). Figure 6 (G and H) supports this claim, as strong negative MAM correlations (−0.85) exist between soil ice and soil liquid water trends at 1.06 m in depth (near the location of the maximum change) for both ssp370 and aerosol mitigation. More generally, significant negative correlations between soil ice and soil liquid trends exist for both December-January-February (DJF) and MAM from ~0.15 to 2 m in depth (fig. S15, A and B). This supports the idea that melting of soil ice leads to an increase in soil liquid (i.e., realizations with a larger decrease in DJF/MAM soil ice have a larger increase in DJF/MAM soil liquid water). This effect is larger in ssp370 due to the enhanced warming (subsurface warming is shown in Fig. 6, E and F). The ssp370 increase in MAM soil liquid water persists at depth (e.g., below 1 m for JJA; Fig. 6C), due in part to seasonal carryover, whereas this is not the case under aerosol mitigation (note S5 and fig. S15, C and D). During JJA [and September-October-November (SON)], both ssp370 and aerosol mitigation feature a decrease in soil liquid water near the surface (Fig. 6, C and D). Although the magnitude of the decrease is similar between ssp370 and aerosol mitigation, drying penetrates deeper under aerosol mitigation (~1.5 m during JJA to 2 m during SON).

To summarize, the larger ssp370 warming melts more soil ice during all seasons (especially MAM), and this leads to a larger increase in soil liquid water at depth, including during JJA, which weakens the decrease in RZSW (RZSW is calculated using soil liquid water down to ~1 m in depth; note S6). This also helps to explain why aerosol mitigation features a larger decrease in RZSW. These results (particularly based on ssp370) are consistent with a prior analysis (*46*), where a vertical soil moisture gradient occurs under global warming.

## DISCUSSION

It is not necessarily surprising that aerosol mitigation yields increased fire activity, as insolation increases due to aerosol reductions will drive significant warming (*31*, *32*, *47*). However, aerosol mitigation yields increases in fire activity in the boreal forest region that are on par with those associated with large GHG increases (i.e., from SSP3-7.0) despite causing only half the warming of the GHG increase. This enhanced FIREC response under aerosol mitigation is associated with deeper drying of the soil column during the NH summertime due to increases in SW_in_ and enhanced evapotranspiration.

We note that the NH boreal forest region FIREC and related climate variable (e.g., surface temperature) trends exhibit considerable spread across realizations under both aerosol mitigation (e.g., Fig. 3) and ssp370 (e.g., fig. S10). For example, NH boreal forest region ANN FIREC trends range from 1.3 to 8.6 kgC km^−2^ day^−1^ decade^−1^ under aerosol mitigation and from 1.8 to 6.5 kgC km^−2^ day^−1^ decade^−1^ under ssp370. This implies that an individual realization of the climate might show relatively small changes in NH boreal forest region fire emissions under aerosol mitigation or ssp370. We emphasize the importance of relatively large ensemble sizes (e.g., such as the 10 used here) to better understand the physical processes underpinning these and other complex changes.

Our conclusions are based on a single climate model, and simulations with additional models are required to corroborate our findings. For example, there are several sources of uncertainty related to the aerosol component of this study. Most importantly is the uncertainty in aerosol effective radiative forcing (ERF), the magnitude of which is ultimately related to surface temperature change and associated climate responses. CESM2 has a relatively strong historical aerosol ERF of −1.34 W m^−2^, but this essentially lies within the 1-sigma uncertainty of −1.09 ± 0.24 W m^−2^ (*48*) based on 20 models from the Coupled Model Intercomparison Project version 6 (CMIP6), with similar results from several other studies (*49*–*51*). Furthermore, in the CESM2 aerosol ERF, this lies within recent observational constraints (*52*) with a 1-sigma confidence range of −1.6 to −0.6 W^−2^. Nonetheless, the relatively strong historical ERF in CESM2 suggests relatively large climate responses to aerosol mitigation in this model (e.g., potentially including FIREC). Another significant source of uncertainty related to the aerosol component of this study is the assumed trajectory of future aerosol/precursor gas emissions. For example, the range of potential global trajectories of aerosol/precursor gas emissions in the shared socioeconomic pathways by the mid-21st century is similar to the growth of emissions over the entire industrial era (*53*). As we have defined aerosol mitigation as the difference between a strong versus weak air quality scenario, our results therefore represent an upper estimate.

We also note that climate models tend to underestimate long-term (e.g., 1960s onward) observed surface solar dimming and subsequent brightening trends as observed by the Global Energy Balance Archive (GEBA) network in some regions, including, for example, over China, Japan, India, and the United States (*54*–*58*). This lack of agreement has persisted through the last few generations of CMIP despite considerable improvements in the representation of aerosol-radiation and in particular aerosol-cloud interactions. This underestimation of observed dimming/brightening trends implies underestimation of the increase in downwelling surface solar radiation under future aerosol mitigation (e.g., Fig. 4A). As this increase in surface solar radiation is important for increasing evapotranspiration and decreasing soil moisture (e.g., RZSW), this in turn implies possible underestimation of the FIREC increase under aerosol mitigation. Although the cause(s) of model underestimation of observed solar dimming/brightening remain outstanding, some studies point to deficient emission inventories (*55*, *57*, *58*). Hence, we reiterate that our study already contains a significant source of uncertainty associated with the assumed trajectory of future aerosol/precursor gas emissions.

Some solar dimming/brightening studies have also pointed to the role of inhomogeneities in the data, e.g., a spurious jump in GEBA surface solar radiation over China in the 1990s is related to widespread changes to the country’s network of instruments (*59*, *60*). We note that the analysis of Quaas *et al.* (*61*), who focused on more recent (2000–2019) satellite data, found good agreement between the Clouds and the Earth’s Radiant Energy System (CERES) clear-sky top-of-the-atmosphere solar radiation trends (in terms of both spatial pattern and magnitude) and those simulated by several CMIP6 models (although CESM2 was not included). For example, over regions with relatively large changes i.e., trends in clear-sky solar ERF greater than 0.05 W m^−2^ year^−1^ (“increasing” regions such as India) or less than −0.05 W m^−2^ year^−1^ (“decreasing” regions including east coast of North America, Europe, and East Asia), CERES yields SW clear sky trends of −0.104 W m^−2^ year^−1^ for the decreasing regions and +0.041 W m^−2^ year^−1^ for the increasing regions. CMIP6 models yields corresponding shortwave clear sky ERF trends of −0.087 and + 0.102 W m^−2^ year^−1^, respectively. In terms of all-sky radiation (which includes cloud effects), CERES shows larger trends but with more noise in the patterns (as compared to clear-sky trends). However, the sign of the changes in the regions where an aerosol signal is expected is consistent between the models and the data.

We acknowledge that most fire models currently have limitations, including, for example, their ability to simulate drought-induced large fires that last multiple days (*24*). Modeling studies that assess future wildfire activity based on the Canadian forest fire weather index (FWI) (*19*) also likely have deficiencies, as the FWI is based on only daily maximum temperature, precipitation, RH, and surface wind and therefore does not take account of other drivers of fire activity, such as live and dead fuel loads, fire suppression, or more complex changes in surface biophysics and hydrology. Thus, considerable uncertainty in future projections of wildfire activity exist, including those discussed here.

As mentioned in Introduction, changes in climate drivers affect fire carbon emissions in our simulations, but fire emissions do not subsequently feedback to impact the climate. These climate change–induced impacts on wildfire activity and their corresponding emissions (including GHGs, aerosols, and precursor gases) are likely to have important impacts on the climate system and air quality. Simulation of these climate-wildfire feedbacks is currently an active area of model development.

The CLM5 model uses RZSW as a proxy for the flammability of fuels (*22*), which affects both the number of ignitions that result in a viable fire, as well as the rate of fire spread. Combined with the Canadian Terrestrial Ecosystem Model (*62*), these two models are unique in their inclusion of a relationship between deep soil moisture status (i.e., RZSW) and fuel flammability (*63*). In contrast, fuel flammability in many fire models is driven by the Nesterov index (derived from daily temperature and RH) or a combination of near-surface soil moisture and RH (*23*). Given that these latter climate variables do not exhibit larger changes under aerosol mitigation (as compared to ssp370), it is likely that the primary mechanism associated with larger increases in NH boreal forest region FIREC in response to aerosol mitigation found here (i.e., larger decreases in RZSW) may not be as pronounced in other models. Certainly, however, increases in SW_in_ will increase temperature and decrease RH and near-surface soil moisture (e.g., Fig. 3), which in turn will increase fuel flammability and likely FIREC in most fire models. Furthermore, experimental evidence suggests that radiation itself is (perhaps unsurprisingly) an important driver of fuel flammability (*64*), suggesting that models that directly estimate the potential evaporation from fuel surfaces may be a more accurate means of predicting flammability. Another study (*65*) highlights the importance of radiation modulation by forest canopies, supporting the overall conclusion of our study, but via the impact of radiation on fuel itself, not via the proxy of soil moisture.

The second mechanism operating in CLM5 illuminated by this study is the relationship in the model between MAM melting soil ice and increasing JJA/SON liquid soil moisture content and fire behavior. The root zone soil moisture index is based on soil moisture potential (*21*), which declines when soils are frozen and no water can be accessed. Thus, this model structure results in thawing soil reducing the flammability of the system. As above, most global fire models, where fuel moisture is based on atmospheric temperature and humidity, or a combination of near-surface soil moisture and RH would likely not feature this response of fire to the phase change of the soil moisture.

Despite these caveats, our results suggest that aerosol mitigation is a significant driver of enhanced wildfire activity in the boreal forest region. The enhanced drying under aerosol mitigation may also have broader implications, including impacts on drought, vegetation, agriculture, and fresh water resources. Regions that likely to experience the largest increase in fires and their associated smoke pollution (i.e., NH boreal forest region and surrounding areas) under aerosol mitigation are not colocated with the regions of large aerosol mitigation (NH subtropics; fig. S1), which could potentially lead to complex cross-national issues. Last, our results support the importance of improved policy-facing evaluations of aerosol-induced near-term climate risks (*53*).

## MATERIALS AND METHODS

### CESM2 description

The CESM2 (*20*) model components use nominal 1° horizontal resolution. For example, the Community Atmosphere Model version 6 has a resolution of 1.25° in longitude and 0.9° in latitude, with 32 vertical levels and a top at 2.26 hPa. The ocean and sea ice models are the Parallel Ocean Program version 2 (*20*) and the CICE Version 5.1.2 (CICE5). Ocean biogeochemistry is simulated with the Marine Biogeochemistry Library. Aerosol processes are represented with the four-mode version of the Modal Aerosol Module (*66*). Prior studies have shown that CESM2 is able to represent aerosol properties (e.g., AOD) reasonably well compared to observations (*67*–*68*).

CESM2 uses the CLM5 (*21*). Photosynthesis and transpiration depend nonlinearly on solar radiation by the light response of stomata. The canopy is treated as two leaves (sunlit and shaded) and the solar radiation in the visible band (<0.7 μm) absorbed by the vegetation is apportioned to the sunlit and shaded leaves. CLM5 explicitly simulates the photosynthetic capacity response to environmental conditions through the Leaf Utilization of Nitrogen for Assimilation module and accounts for how nitrogen availability affects plant productivity through the Fixation and Uptake of Nitrogen module (*21*). Soil water is predicted from a multilayer model, in which vertical soil moisture transport is governed by infiltration, surface and subsurface runoff, gradient diffusion, gravity, canopy transpiration through root extraction, and interactions with ground water.

CLM5 is not a Dynamic Global Vegetation Model and thus does not simulate changes in the distribution or type of vegetation in response to climate change. Vegetation distributions, however, do change through time (see below) based on the SSP3-7.0 land use time series file. CLM5, however, does simulate changes in vegetation physiology (e.g., photosynthesis, transpiration, and stomatal conductance) and vegetation state such as leaf area index (*21*).

CLM’s fire module parameterizes burned area, carbon emissions, and biomass burning aerosol/precursor gas emissions (*22*–*24*). Although fire trace gases and aerosol emissions can be simulated, our CESM2 simulations use prescribed fire aerosol emissions. Four types of fires are represented, including agricultural fires in croplands, peat fires, deforestation fires in tropical closed forests, and wildfires (i.e., nonpeat fires outside of croplands and tropical closed forests). Burned area depends on ignitions, fire suppression, fuel load, and fuel combustibility (*22*, *69*, *70*). Natural ignition is parameterized as a function of lightning frequency; anthropogenic ignition (and suppression) is parameterized as a function of both population density and gross domestic product. Fuel load is determined by the amount of all types of vegetation and litter present in a grid cell. Fuel combustibility is a function of surface soil wetness (i.e., volumetric soil moisture relative to that at saturation), RH, and surface air temperature, and fire spread depends on wind speed, RH, and soil moisture (here, root zone soil wetness). RZSW is a metric weighted toward the upper layers of soil via the root distribution function and is used in CLM5 as a proxy for fuel combustibility (*21*).

Note that human effects on ignition and suppression affect both ssp370 and ssp370-126aer (e.g., due to population changes), but not the difference (i.e., aerosol mitigation). However, human ignition/suppression effects on fires in the boreal forest region under ssp370 will likely be small due to minimal human population and relatively small 2015–2060 SSP3-7.0 population trends (fig. S16). Similarly, although our CESM2 simulations feature changes in land use/land change from SSP3-7.0, these changes do not affect aerosol mitigation (for the same reason as above). Land use/land change also likely has minimal impacts on FIREC trends in ssp370, as the NH boreal forest region is not a region subject to significant anthropogenic land use/land change disturbances. For example, the 2015–2060 trends in crop, tree, and grass fraction in the NH boreal forest region are all relatively small and not significant (fig. S17). We also reiterate that our ssp370 NH boreal forest region FIREC trends (3.89 ± 0.86 kgC km^−2^ day^−1^ decade^−1^) are very similar to those from CESM2 GHG-only single forcing runs (3.70 ± 1.30 kgC km^−2^ day^−1^ decade^−1^). The GHG-only forced simulations feature fixed land use/land change (however, it is less clear if population is also fixed). The similar FIREC trends between our ssp370 simulations and CESM2 GHG-only simulations imply minimal effects from land use/land change. Nonetheless, we acknowledge that we cannot completely rule out potential impacts of population and land use change on FIREC trends in our baseline ssp370 experiment.

The CLM fire scheme has been previously evaluated in both uncoupled and coupled versions (*22*, *70*–*73*), and it has been compared with other fire models within the Fire Modeling Intercomparison Project (FireMIP) (*24*). The CLM fire module can reasonably reproduce the observed amount, spatial pattern, and seasonality of global fires; the interannual variability in global fires; and the present-day fire-population relationship. More recently (*17*), fire carbon emissions from CESM2 (WACCM6) were evaluated against two satellite-based fire emission inventories, including FINNv2.5 (Fire INventory from NCAR version 2.5) (*74*) and GFED4.1 s (Global Fire Emissions Database, version 4.1 s) (*75*). The annual total fire carbon emissions and spatial distributions agree well with those from FINNv2.5 and GFED4.1 s. For example, the 2015–2019 annual total fire carbon emissions from CESM2 (WACCM6) simulations is 2.5 PgC year^−1^, which falls in between GFED4.1 s at 2.0 PgC year^−1^ and FINNv2.5 at 3.8 PgC year^−1^. More recently, Allen *et al.* (*25*) showed that CMIP6 models can reasonably reproduce observed 2002–2021 FIREC climatologies, and CESM2 is one of the better models in the NH boreal regions, particularly over Canada and central/north Asia (and the United States). A more comprehensive evaluation, including trends and the use of the newly released GFED5 (*15*), will be the subject of future investigation.

### CESM2 experiments

CESM2 21st century simulations are based on anthropogenic emissions and land use/land change from the Shared Socioeconomic Pathway 3-7.0 (SSP3-7.0) (*27*–*29*). Our baseline experiment is ssp370, which comes from archived simulations performed under the CESM2 Large Ensemble (CESM2-LE) Project (*33*). The ssp370 CESM2-LE simulations are an extension of the corresponding historical (1850–2014) simulations, i.e., CESM2-LE simulations span 1850–2100, with SSP3-7.0 emissions used from 2015 onward. Specifically, we use 10 of the macroperturbation runs (i.e., each ensemble member is initialized from a different year in the preindustrial control simulation) that use an 11-year running mean filter to smooth the CMIP6 biomass burning emissions, including members 1011-001, 1031-002, 1051-003, 1071-004, 1091-005, 1111-006, 1131-007, 1151-008, 1171-009, and 1191-010. This smoothing reduces the variability in biomass burning fluxes over 1990–2020 (satellite data are use from 1997–2014, which contains more internannual variability than data sources used before 1997 and after 2014). Prior analyses (*76*, *77*) have shown that using the default CMIP6 biomass burning emissions (with larger variability over the satellite-era) affects the large-scale climate in CESM2 (largely from 1990–2010), including accelerated loss of September Arctic sea ice through summertime aerosol-cloud interactions. We also note that data for our 2000–2014 climatologies comes from the CESM2-LE historical simulations (using the same 10 ensemble members as above).

The CESM2 simulations performed here (i.e., ssp370-126aer), as part of the RAMIP (*26*), were initialized from the end of the CESM2-LE historical simulations. These simulations span 2015–2060 and include 10 ensemble members. They use an identical setup to ssp370, including prescribed concentrations of well-mixed GHGs, ozone, natural emissions (e.g., biogenic emissions), and land use changes from the SSP3-7.0 pathway—but take global anthropogenic aerosol and precursor emissions (SO_2_, SO_4_, black carbon, primary organic matter, and anthropogenic SOA precursors) from all anthropogenic sources (e.g., shipping, aircraft, agriculture, residential, energy, industrial, etc.) under the SSP1-2.6 pathway. This includes biomass burning emissions (e.g., forest and grassland burning).

We acknowledge that labeling biomass burning emissions as “anthropogenic” is a gray area, and moreover, including them in our experimental design is somewhat physically unrealistic. For example, our prescribed biomass burning aerosols from SSP3-7.0 or SSP1-2.6 could affect wildfire activity and the FIREC trends discussed here. However, the aforementioned evaluation of the CESM2 single forcing experiments (*34*) showed relatively small, nonsignificant changes in FIREC under prescribed SSP3-7.0 biomass burning aerosol and precursor gas emissions. Furthermore, canonical model experimental design (e.g., detection and attribution MIP) lumps both industrial and biomass burning aerosols emissions together (outside of a few exceptions, including the CESM2 single forcing experiments). Although including the prescribed SSP3-7.0 or SSP1-2.6 biomass burning emissions in our simulations may add additional uncertainty to our results, we suggest this is minimal. This statement is based on the fact the bulk of historical (2014 relative to 1850) total aerosol ERF (*78*) at −1.01 ± 0.25 W m^−2^ comes from SO_2_ at −1.03 ± 0.37 W m^−2^. The organic carbon (OC) and black carbon (BC) ERFs however, are much smaller (and have offsetting effects) at −0.25 ± 0.09 and 0.15 ± 0.17 W m^−2^, respectively. As wildfire emissions largely emit carbonaceous aerosols (BC and OC), the bulk of the warming and climate responses under our aerosol mitigation signal are largely driven by nonbiomass burning aerosol emission reductions. This is supported by the fact that total SO_2_ emissions in SSP1-2.6 decrease from 100.8 MtSO_2_ year^−1^ in 2015 to 22.0 MtSO_2_ year^−1^ in 2060. The corresponding contribution of forest burning to SO_2_ emissions is very small at 0.67 MtSO_2_ year^−1^ in 2015 and 0.23 MtSO_2_ year^−1^ in 2060 (i.e., <1% of total SO_2_ emissions). Thus, although forest burning SO_2_ emissions (the dominant aerosol that will drive future warming and associated climate changes) decrease under SSP1-2.6, their relative contribution to the total SO_2_ decrease is very small at <1%. The corresponding calculation for OC and BC shows a larger relative contribution of forest burning to the total decrease in both species at 22% and 6%, respectively. However, as mentioned above, these will have competing effects on climate (OC reductions will warm whereas BC reductions will cool). In addition, the warming effects of future OC reductions will be much smaller than those associated with future SO_2_ reductions (i.e., as supported by the much weaker OC ERF as compared to the SO_2_ ERF).

SSP3-7.0 features large increases in GHGs through the 21st century but relatively small changes in aerosol and precursor gas emissions (generally weak increases by mid-century, followed by decreases, with small overall changes by 2100). For example, atmospheric CO_2_ concentrations increase to ~900 parts per million by 2100 (>100% increase relative to 2015); CH_4_ concentrations increase to 3400 parts per billion (~80% increase relative to 2015). In contrast, SSP1-2.6 features large reductions in aerosol and precursor gas emissions. For example, by mid-century, global BC emissions under SSP1-2.6 decrease from ~8 Tg year^−1^ in 2015 to ~2 Tg year^−1^ in 2050. In contrast, under SSP3-7.0, global BC emissions increase slightly to ~9 Tg year^−1^ by mid-century. Similarly, global SO_2_ emissions under SSP1-2.6 decrease from ~100 Tg year^−1^ in 2015 to ~30 Tg year^−1^ in 2050. Under SSP3-7.0, global SO_2_ emissions remain relatively fixed up to the mid-century. Thus, taking the difference of the two experiments (ssp370-126aer-ssp370) yields the aerosol mitigation signal, as represented by the difference between the weak air quality control SSP3-7.0 pathway relative to the strong air quality control SSP1-2.6 pathway. We acknowledge that combining the two pathways (i.e., large increases in GHGs consistent with SSP3-7.0 with simultaneous large reductions in aerosol/precursor gases under SSP1-2.6) is unlikely to occur in reality and that our results (e.g. the magnitude of the FIREC increase) likely represent an upper bound as the baseline scenario (SSP3-7.0) contains the highest levels of aerosol/precursor gas emissions.

### Data processing and statistics

We use monthly mean CESM2 data at the model’s native spatial resolution (1.25° longitude by 0.9° latitude). Climate responses are estimated as the 2015–2060 slope using a standard least-squares regression, and significance is based on a standard two-tailed *t* test. Significance of correlations (*r*) is estimated from a two-tailed *t* test as: $t=\frac{r}{\sqrt{\frac{1-r^{2}}{n-2}}}$ , with *n* − 2 degrees of freedom. Here, *n* is either the number of years (for a correlation over time) or the number of ensemble members (i.e., 46 years and 10 realizations, respectively). Quoted trend uncertainties in the manuscript are estimated as the 90% confidence interval according to $\frac{1.65\times \sigma }{\sqrt{n-1}}$ , where σ is the SD across the trends and *n* is the number of trends (i.e., 10). Percent change (relative to 2000–2014 climatology) is estimated from the slope of the 2015–2060 trend line, multiplied by the 46 years and divided by the 2000–2014 climatology, and subsequently multiplied by 100.

Aerosol amplification (e.g., Fig. 1, E and F) is quantified on the basis of the percent change of the trends, which is estimated as [(aerosol mitigation trend/ssp370 trend) ×100]. Depending on the climate variable, this could include aerosol amplification of positive trends (e.g., FIREC) or aerosol amplification of negative trends (e.g., RZSW). However, under either circumstance, only two conditions are plotted. Aerosol amplification of a positive trend is graphically displayed in a dark red color when (i) both the aerosol mitigation and ssp370 trends are positive and the percent change is greater than 100% or (ii) the aerosol mitigation trend is positive, but the ssp370 trend is negative. The former shows regions where both aerosol mitigation and ssp370 yield increases, but larger increases occur under aerosol mitigation. The latter shows regions where aerosol mitigation drives an increase whereas ssp370 yields a decrease. Similarly, aerosol amplification of a negative trend is graphically displayed as a dark blue color when (i) both the aerosol mitigation and ssp370 trends are negative and the percent change is greater than 100% or (ii) the aerosol mitigation trend is negative, but the ssp370 trend is positive. The former shows regions where both aerosol mitigation and ssp370 yield decreases, but larger decreases occur under aerosol mitigation. The latter shows regions where aerosol mitigation drives a decrease whereas ssp370 yields an increase. Thus, any shaded region (whether red or blue) shows amplification of the trend under aerosol mitigation.

The NH boreal forest region is the world’s largest terrestrial biome, consisting of deciduous trees and conifers. It is a region characterized by high vegetation carbon content and tree fraction, as well as a region that experiences wildfires. Here, we define the NH boreal forest region as all grid boxes over land from 45°N to 90°N with at least 50% tree fraction (Fig. 1G). It comprises 27% of the land area from 45°N to 90°N. We note that our definition of the boreal forest region does not include some areas typically included in the definition of “boreal,” including the taiga-dominated regions of northern Canada, most of Alaska, and northern Siberia.

## Supplementary Materials

### This PDF file includes:

Notes S1 to S6 Figs. S1 to S17

## References

[1] J. C. Liu, L. J. Mickley, M. P. Sulprizio, F. Dominici, X. Yue, K. Ebisu, G. B. Anderson, R. F. A. Khan, M. A. Bravo, M. L. Bell, Particulate air pollution from wildfires in the Western US under climate change. Clim. Change 138, 655–666 (2016). 28642628 10.1007/s10584-016-1762-6 PMC5476308

[2] M. Burke, A. Driscoll, S. Heft-Neal, J. Xue, J. Burney, M. Wara, The changing risk and burden of wildfire in the United States. Proc. Natl. Acad. Sci. U.S.A. 118, e2011048118 (2021). 33431571 10.1073/pnas.2011048118 PMC7812759

[3] Y. Xie, M. Lin, B. Decharme, C. Delire, L. W. Horowitz, D. M. Lawrence, F. Li, R. Séférian, Tripling of western US particulate pollution from wildfires in a warming climate. Proc. Natl. Acad. Sci. U.S.A. 119, e2111372119 (2022). 35344431 10.1073/pnas.2111372119 PMC9168465

[4] O. Pechony, D. T. Shindell, Driving forces of global wildfires over the past millennium and the forthcoming century. Proc. Natl. Acad. Sci. U.S.A. 107, 19167–19170 (2010). 20974914 10.1073/pnas.1003669107 PMC2984177

[5] J. T. Abatzoglou, A. P. Williams, R. Barbero, Global emergence of anthropogenic climate change in fire weather indices. Geophys. Res. Lett. 46, 326–336 (2019).

[6] United Nations Environment Programme, Spreading like Wildfire—The Rising Threat of Extraordinary Landscape Fires (UN, 2022).

[7] M. S. Balshi, A. D. McGuire, P. Duffy, M. Flannigan, J. Walsh, J. Melillo, Assessing the response of area burned to changing climate in western boreal North America using a Multivariate Adaptive Regression Splines (MARS) approach. Glob. Chang. Biol. 15, 578–600 (2009).

[8] M. D. Flannigan, M. A. Krawchuk, W. J. de Groot, B. M. Wotton, L. M. Gowman, Implications of changing climate for global wildland fire. Int. J. Wildland Fire 18, 483–507 (2009).

[9] M. P. Calef, A. Varvak, A. D. McGuire, F. S. Chapin III, K. B. Reinhold, Recent changes in annual area burned in interior Alaska: The impact of fire management. Earth Interact. 19, 1–17 (2015).

[10] C. C. Hanes, X. Wang, P. Jain, M.-A. Parisien, J. M. Little, M. D. Flannigan, Fire-regime changes in Canada over the last half century. Can. J. For. Res. 49, 256–269 (2019).

[11] B. Amiro, A. Cantin, M. Flannigan, W. de Groot, Future emissions from Canadian boreal forest fires. Can. J. For. Res. 39, 383–395 (2009).

[12] M. D. Flannigan, K. A. Logan, B. D. Amiro, W. R. Skinner, B. J. Stocks, Future area burned in Canada. Clim. Change 72, 1–16 (2005).

[13] C. Y. Park, K. Takahashi, F. Li, J. Takakura, S. Fujimori, T. Hasegawa, A. Ito, D. K. Lee, W. Thiery, Impact of climate and socioeconomic changes on fire carbon emissions in the future: Sustainable economic development might decrease future emissions. Glob. Environ. Change 80, 102667 (2023).

[14] M. T. Lund, K. Nordling, A. B. Gjelsvik, B. H. Samset, The influence of variability on fire weather conditions in high latitude regions under present and future global warming. Environ. Res. Commun. 5, 065016 (2023).

[15] Y. Chen, J. Hall, D. van Wees, N. Andela, S. Hantson, L. Giglio, G. R. van der Werf, D. C. Morton, J. T. Randerson, Multi-decadal trends and variability in burned area from the fifth version of the Global Fire Emissions Database (GFED5). Earth Syst. Sci. Data 15, 5227–5259 (2023).

[16] X. J. Walker, B. M. Rogers, S. Veraverbeke, J. F. Johnstone, J. L. Baltzer, K. Barrett, L. Bourgeau-Chavez, N. J. Day, W. J. de Groot, C. M. Dieleman, S. Goetz, E. Hoy, L. K. Jenkins, E. S. Kane, M. A. Parisien, S. Potter, E. A. G. Schuur, M. Turetsky, E. Whitman, M. C. Mack, Fuel availability not fire weather controls boreal wildfire severity and carbon emissions. Nat. Clim. Chang. 10, 1130–1136 (2020).

[17] W. Tang, S. Tilmes, D. M. Lawrence, F. Li, C. He, L. K. Emmons, R. R. Buchholz, L. Xia, Impact of solar geoengineering on wildfires in the 21st century in CESM2/WACCM6. Atmos. Chem. Phys. 23, 5467–5486 (2023).

[18] D. Touma, S. Stevenson, F. Lehner, S. Coats, Human-driven greenhouse gas and aerosol emissions cause distinct regional impacts on extreme fire weather. Nat. Commun. 12, 212 (2021). 33431844 10.1038/s41467-020-20570-w PMC7801713

[19] C. E. Van Wagner, Structure of the Canadian Forest Fire Weather Index, (Environment Canada, Canadain Forestry Service, Petawawa Forest Experiment Station, 1974).

[20] G. Danabasoglu, J.-F. Lamarque, J. Bacmeister, D. A. Bailey, A. K. DuVivier, J. Edwards, L. K. Emmons, J. Fasullo, R. Garcia, A. Gettelman, C. Hannay, M. M. Holland, W. G. Large, P. H. Lauritzen, D. M. Lawrence, J. T. M. Lenaerts, K. Lindsay, W. H. Lipscomb, M. J. Mills, R. Neale, K. W. Oleson, B. Otto-Bliesner, A. S. Phillips, W. Sacks, S. Tilmes, L. van Kampenhout, M. Vertenstein, A. Bertini, J. Dennis, C. Deser, C. Fischer, B. Fox-Kemper, J. E. Kay, D. Kinnison, P. J. Kushner, V. E. Larson, M. C. Long, S. Mickelson, J. K. Moore, E. Nienhouse, L. Polvani, P. J. Rasch, W. G. Strand, The community earth system model version 2 (CESM2). J. Adv. Model. Earth Sys. 12, e2019MS001916 (2020).

[21] D. M. Lawrence, R. A. Fisher, C. D. Koven, K. W. Oleson, S. C. Swenson, G. Bonan, N. Collier, B. Ghimire, L. van Kampenhout, D. Kennedy, E. Kluzek, P. J. Lawrence, F. Li, H. Li, D. Lombardozzi, W. J. Riley, W. J. Sacks, M. Shi, M. Vertenstein, W. R. Wieder, C. Xu, A. A. Ali, A. M. Badger, G. Bisht, M. van den Broeke, M. A. Brunke, S. P. Burns, J. Buzan, M. Clark, A. Craig, K. Dahlin, B. Drewniak, J. B. Fisher, M. Flanner, A. M. Fox, P. Gentine, F. Hoffman, G. Keppel-Aleks, R. Knox, S. Kumar, J. Lenaerts, L. R. Leung, W. H. Lipscomb, Y. Lu, A. Pandey, J. D. Pelletier, J. Perket, J. T. Randerson, D. M. Ricciuto, B. M. Sanderson, A. Slater, Z. M. Subin, J. Tang, R. Q. Thomas, M. Val Martin, X. Zeng, The community land model version 5: Description of new features, benchmarking, and impact of forcing uncertainty. J. Adv. Model. Earth Sys. 11, 4245–4287 (2019).

[22] F. Li, X. D. Zeng, S. Levis, A process-based fire parameterization of intermediate complexity in a Dynamic Global Vegetation Model. Biogeosciences 9, 2761–2780 (2012).

[23] S. S. Rabin, J. R. Melton, G. Lasslop, D. Bachelet, M. Forrest, S. Hantson, J. O. Kaplan, F. Li, S. Mangeon, D. S. Ward, C. Yue, V. K. Arora, T. Hickler, S. Kloster, W. Knorr, L. Nieradzik, A. Spessa, G. A. Folberth, T. Sheehan, A. Voulgarakis, D. I. Kelley, I. C. Prentice, S. Sitch, S. Harrison, A. Arneth, The Fire Modeling Intercomparison Project (FireMIP), phase 1: Experimental and analytical protocols with detailed model descriptions. Geosci. Model Dev. 10, 1175–1197 (2017).

[24] F. Li, M. Val Martin, M. O. Andreae, A. Arneth, S. Hantson, J. W. Kaiser, G. Lasslop, C. Yue, D. Bachelet, M. Forrest, E. Kluzek, X. Liu, S. Mangeon, J. R. Melton, D. S. Ward, A. Darmenov, T. Hickler, C. Ichoku, B. I. Magi, S. Sitch, G. R. van der Werf, C. Wiedinmyer, S. S. Rabin, Historical (1700–2012) global multi-model estimates of the fire emissions from the Fire Modeling Intercomparison Project (FireMIP). Atmos. Chem. Phys. 19, 12545–12567 (2019).

[25] R. J. Allen, J. Gomez, L. W. Horowitz, E. Shevliakova, Enhanced future vegetation growth with elevated carbon dioxide concentrations could increase fire activity. Commun. Earth Environ. 5, 54 (2024).

[26] L. J. Wilcox, R. J. Allen, B. H. Samset, M. A. Bollasina, P. T. Griffiths, J. M. Keeble, M. T. Lund, R. Makkonen, J. Merikanto, D. O’Donnell, D. J. Paynter, G. G. Persad, S. T. Rumbold, T. Takemura, K. Tsigaridis, S. Undorf, D. M. Westervelt, The Regional Aerosol Model Intercomparison Project (RAMIP). Geosci. Model Dev. Discuss. 2022, 1–40 (2022).

[27] B. C. O’Neill, C. Tebaldi, D. P. van Vuuren, V. Eyring, P. Friedlingstein, G. Hurtt, R. Knutti, E. Kriegler, J.-F. Lamarque, J. Lowe, G. A. Meehl, R. Moss, K. Riahi, B. M. Sanderson, The scenario model intercomparison project (ScenarioMIP) for CMIP6. Geosci. Model Dev. 9, 3461–3482 (2016).

[28] S. Rao, Z. Klimont, S. J. Smith, R. Van Dingenen, F. Dentener, L. Bouwman, K. Riahi, M. Amann, B. L. Bodirsky, D. P. van Vuuren, L. Aleluia Reis, K. Calvin, L. Drouet, O. Fricko, S. Fujimori, D. Gernaat, P. Havlik, M. Harmsen, T. Hasegawa, C. Heyes, J. Hilaire, G. Luderer, T. Masui, E. Stehfest, J. Strefler, S. van der Sluis, M. Tavoni, Future air pollution in the Shared Socio-economic Pathways. Glob. Environ. Change 42, 346–358 (2017).

[29] K. Riahi, D. P. van Vuuren, E. Kriegler, J. Edmonds, B. C. O’Neill, S. Fujimori, N. Bauer, K. Calvin, R. Dellink, O. Fricko, W. Lutz, A. Popp, J. C. Cuaresma, S. KC, M. Leimbach, L. Jiang, T. Kram, S. Rao, J. Emmerling, K. Ebi, T. Hasegawa, P. Havlik, F. Humpenöder, L. A. Da Silva, S. Smith, E. Stehfest, V. Bosetti, J. Eom, D. Gernaat, T. Masui, J. Rogelj, J. Strefler, L. Drouet, V. Krey, G. Luderer, M. Harmsen, K. Takahashi, L. Baumstark, J. C. Doelman, M. Kainuma, Z. Klimont, G. Marangoni, H. Lotze-Campen, M. Obersteiner, A. Tabeau, M. Tavoni, The Shared Socioeconomic Pathways and their energy, land use, and greenhouse gas emissions implications: An overview. Glob. Environ. Change 42, 153–168 (2017).

[30] W. J. Collins, J.-F. Lamarque, M. Schulz, O. Boucher, V. Eyring, M. I. Hegglin, A. Maycock, G. Myhre, M. Prather, D. Shindell, S. J. Smith, AerChemMIP: Quantifying the effects of chemistry and aerosols in CMIP6. Geosci. Model Dev. 10, 585–607 (2017).

[31] R. J. Allen, S. Turnock, P. Nabat, D. Neubauer, U. Lohmann, D. Olivié, N. Oshima, M. Michou, T. Wu, J. Zhang, T. Takemura, M. Schulz, K. Tsigaridis, S. E. Bauer, L. Emmons, L. Horowitz, V. Naik, T. van Noije, T. Bergman, J.-F. Lamarque, P. Zanis, I. Tegen, D. M. Westervelt, P. Le Sager, P. Good, S. Shim, F. O’Connor, D. Akritidis, A. K. Georgoulias, M. Deushi, L. T. Sentman, J. G. John, S. Fujimori, W. J. Collins, Climate and air quality impacts due to mitigation of non-methane near-term climate forcers. Atmos. Chem. Phys. 20, 9641–9663 (2020).

[32] R. J. Allen, L. W. Horowitz, V. Naik, N. Oshima, F. M. O’Connor, S. Turnock, S. Shim, P. L. Sager, T. van Noije, K. Tsigaridis, S. E. Bauer, L. T. Sentman, J. G. John, C. Broderick, M. Deushi, G. A. Folberth, S. Fujimori, W. J. Collins, Significant climate benefits from near-term climate forcer mitigation in spite of aerosol reductions. Environ. Res. Lett. 16, 034010 (2021).

[33] K. B. Rodgers, S.-S. Lee, N. Rosenbloom, A. Timmermann, G. Danabasoglu, C. Deser, J. Edwards, J.-E. Kim, I. R. Simpson, K. Stein, M. F. Stuecker, R. Yamaguchi, T. Bódai, E.-S. Chung, L. Huang, W. M. Kim, J.-F. Lamarque, D. L. Lombardozzi, W. R. Wieder, S. G. Yeager, Ubiquity of human-induced changes in climate variability. Earth Syst. Dyn. 12, 1393–1411 (2021).

[34] I. R. Simpson, N. Rosenbloom, G. Danabasoglu, C. Deser, S. G. Yeager, C. S. McCluskey, R. Yamaguchi, J.-F. Lamarque, S. Tilmes, M. J. Mills, K. B. Rodgers, The CESM2 single-forcing large ensemble and comparison to CESM1: Implications for experimental design. J. Clim. 36, 5687–5711 (2023).

[35] K. W. Oleson, D. M. Lawrence, G. B. Bonan, B. Drewniak, M. Huang, C. D. Koven, S. Levis, F. Li, W. J. Riley, Z. M. Subin, S. C. Swenson, P. E. Thornton, A. Bozbiyik, R. Fisher, C. L. Heald, E. Kluzek, J-F. Lamarque, P. J. Lawrence, L. R. Leung, W. Lipscomb, S. Muszala, D. M. Ricciuto, W. Sacks, Y. Sun, J. Tang, Z-L. Yang, Technical description of version 4.5 of the Community Land Model (CLM) (NCAR Technical Note No. NCAR/TN-503+ STR) (National Center for Atmospheric Research, 2013); doi: 10.5065/D6RR1W7M.

[36] G. Lasslop, S. Kloster, Impact of fuel variability on wildfire emission estimates. Atmos. Envion. 121, 93–102 (2015).

[37] S. I. Seneviratne, T. Corti, E. L. Davin, M. Hirschi, E. B. Jaeger, I. Lehner, B. Orlowsky, A. J. Teuling, Investigating soil moisture–climate interactions in a changing climate: A review. Earth Sci. Rev. 99, 125–161 (2010).

[38] J. M. C. Denissen, A. J. Teuling, A. J. Pitman, S. Koirala, M. Migliavacca, W. Li, M. Reichstein, A. J. Winkler, C. Zhan, R. Orth, Widespread shift from ecosystem energy to water limitation with climate change. Nat. Clim. Chang. 12, 677–684 (2022).

[39] L. Gu, D. Baldocchi, S. B. Verma, T. A. Black, T. Vesala, E. M. Falge, P. R. Dowty, Advantages of diffuse radiation for terrestrial ecosystem productivity. J. Geophys. Res. Atmos. 107, ACL 2–1–ACL 2–23 (2002).

[40] X. Pedruzo-Bagazgoitia, H. G. Ouwersloot, M. Sikma, C. C. van Heerwaarden, C. M. J. Jacobs, J. V.-G. de Arellano, Direct and diffuse radiation in the shallow cumulus–vegetation system: Enhanced and decreased evapotranspiration regimes. J. Hydrometeorol. 18, 1731–1748 (2017).

[41] E. A. Ainsworth, S. P. Long, What have we learned from 15 years of free-air CO_2_ enrichment (FACE)? A meta-analytic review of the responses of photosynthesis, canopy properties and plant production to rising CO_2_. New Phytol. 165, 351–372 (2005). 15720649 10.1111/j.1469-8137.2004.01224.x

[42] E. A. Ainsworth, A. Rogers, The response of photosynthesis and stomatal conductance to rising [CO_2_]: Mechanisms and environmental interactions. Plant Cell Environ. 30, 258–270 (2007). 17263773 10.1111/j.1365-3040.2007.01641.x

[43] V. Haverd, B. Smith, J. G. Canadell, M. Cuntz, S. Mikaloff-Fletcher, G. Farquhar, W. Woodgate, P. R. Briggs, C. M. Trudinger, Higher than expected CO_2_ fertilization inferred from leaf to global observations. Glob. Chang. Biol. 26, 2390–2402 (2020). 32017317 10.1111/gcb.14950 PMC7154678

[44] C. Chen, W. J. Riley, I. C. Prentice, T. F. Keenan, CO_2_ fertilization of terrestrial photosynthesis inferred from site to global scales. Proc. Natl. Acad. Sci. U.S.A. 119, e2115627119 (2022). 35238668 10.1073/pnas.2115627119 PMC8915860

[45] A. L. S. Swann, F. M. Hoffman, C. D. Koven, J. T. Randerson, Plant responses to increasing CO_2_ reduce estimates of climate impacts on drought severity. Proc. Natl. Acad. Sci. U.S.A. 113, 10019–10024 (2016). 27573831 10.1073/pnas.1604581113 PMC5018756

[46] A. Berg, J. Sheffield, P. C. D. Milly, Divergent surface and total soil moisture projections under global warming. Geophys. Res. Lett. 44, 236–244 (2017).

[47] B. H. Samset, M. Sand, C. J. Smith, S. E. Bauer, P. M. Forster, J. S. Fuglestvedt, S. Osprey, C.-F. Schleussner, Climate impacts from a removal of anthropogenic aerosol emissions. Geophys. Res. Lett. 45, 1020–1029 (2018). 32801404 10.1002/2017GL076079 PMC7427631

[48] M. D. Zelinka, C. J. Smith, Y. Qin, K. E. Taylor, Comparison of methods to estimate aerosol effective radiative forcings in climate models. Atmos. Chem. Phys. 23, 8879–8898 (2023).

[49] C. J. Smith, R. J. Kramer, G. Myhre, K. Alterskjær, W. Collins, A. Sima, O. Boucher, J.-L. Dufresne, P. Nabat, M. Michou, S. Yukimoto, J. Cole, D. Paynter, H. Shiogama, F. M. O’Connor, E. Robertson, A. Wiltshire, T. Andrews, C. Hannay, R. Miller, L. Nazarenko, A. Kirkevåg, D. Olivié, S. Fiedler, A. Lewinschal, C. Mackallah, M. Dix, R. Pincus, P. M. Forster, Effective radiative forcing and adjustments in CMIP6 models. Atmos. Chem. Phys. 20, 9591–9618 (2020).

[50] C. J. Smith, G. R. Harris, M. D. Palmer, N. Bellouin, W. Collins, G. Myhre, M. Schulz, J.-C. Golaz, M. Ringer, T. Storelvmo, P. M. Forster, Energy budget constraints on the time history of aerosol forcing and climate sensitivity. J. Geophys. Res. Atmos. 126, e2020JD033622 (2021).

[51] A. Kalisoras, A. K. Georgoulias, D. Akritidis, R. J. Allen, V. Naik, C. Kuo, S. Szopa, P. Nabat, D. Olivié, T. van Noije, P. Le Sager, D. Neubauer, N. Oshima, J. Mulcahy, L. W. Horowitz, P. Zanis, Decomposing the effective radiative forcing of anthropogenic aerosols based on CMIP6 earth system models (EGUsphere, 2023), pp. 1–38.

[52] N. Bellouin, J. Quaas, E. Gryspeerdt, S. Kinne, P. Stier, D. Watson-Parris, O. Boucher, K. S. Carslaw, M. Christensen, A.-L. Daniau, J.-L. Dufresne, G. Feingold, S. Fiedler, P. Forster, A. Gettelman, J. M. Haywood, U. Lohmann, F. Malavelle, T. Mauritsen, D. T. McCoy, G. Myhre, J. Mülmenstädt, D. Neubauer, A. Possner, M. Rugenstein, Y. Sato, M. Schulz, S. E. Schwartz, O. Sourdeval, T. Storelvmo, V. Toll, D. Winker, B. Stevens, Bounding global aerosol radiative forcing of climate change. Rev. Geophys. 58, e2019RG000660 (2020). 10.1029/2019RG000660 PMC7384191 32734279

[53] G. Persad, B. H. Samset, L. J. Wilcox, R. J. Allen, M. A. Bollasina, B. B. B. Booth, C. Bonfils, T. Crocker, M. Joshi, M. T. Lund, K. Marvel, J. Merikanto, K. Nordling, S. Undorf, D. P. van Vuuren, D. M. Westervelt, A. Zhao, Rapidly evolving aerosol emissions are a dangerous omission from near-term climate risk assessments. Environ. Res. Climate 2, 032001 (2023).

[54] M. Wild, Enlightening global dimming and brightening. Bull. Am. Meteorol. Soc. 93, 27–37 (2012).

[55] R. J. Allen, J. R. Norris, M. Wild, Evaluation of multidecadal variability in CMIP5 surface solar radiation and inferred underestimation of aerosol direct effects over Europe China, Japan, and India. J. Geophys. Res. Atmos. 118, 6311–6336 (2013).

[56] T. Storelvmo, U. K. Heede, T. Leirvik, P. C. B. Phillips, P. Arndt, M. Wild, Lethargic response to aerosol emissions in current climate models. Geophys. Res. Lett. 45, 9814–9823 (2018).

[57] K. O. Moseid, M. Schulz, T. Storelvmo, I. R. Julsrud, D. Olivié, P. Nabat, M. Wild, J. N. S. Cole, T. Takemura, N. Oshima, S. E. Bauer, G. Gastineau, Bias in CMIP6 models as compared to observed regional dimming and brightening. Atmos. Chem. Phys. 20, 16023–16040 (2020).

[58] I. R. Julsrud, T. Storelvmo, M. Schulz, K. O. Moseid, M. Wild, Disentangling Aerosol and Cloud Effects on Dimming and Brightening in Observations and CMIP6. J. Geophys. Res. Atmos. 127, e2021JD035476 (2022).

[59] Y. Wang, M. Wild, A new look at solar dimming and brightening in China. Geophys. Res. Lett. 43, 11,777–11,785 (2016).

[60] S. Yang, X. L. Wang, M. Wild, Homogenization and Trend Analysis of the 1958–2016 In Situ Surface Solar Radiation Records in China. J. Clim. 31, 4529–4541 (2018).

[61] J. Quaas, H. Jia, C. Smith, A. L. Albright, W. Aas, N. Bellouin, O. Boucher, M. Doutriaux-Boucher, P. M. Forster, D. Grosvenor, S. Jenkins, Z. Klimont, N. G. Loeb, X. Ma, V. Naik, F. Paulot, P. Stier, M. Wild, G. Myhre, M. Schulz, Robust evidence for reversal of the trend in aerosol effective climate forcing. Atmos. Chem. Phys. 22, 12221–12239 (2022).

[62] V. K. Arora, G. J. Boer, Fire as an interactive component of dynamic vegetation models. J. Geophys. Res. Biogeo. 110, 10.1029/2005JG000042 (2005).

[63] S. Hantson, A. Arneth, S. P. Harrison, D. I. Kelley, I. C. Prentice, S. S. Rabin, S. Archibald, F. Mouillot, S. R. Arnold, P. Artaxo, D. Bachelet, P. Ciais, M. Forrest, P. Friedlingstein, T. Hickler, J. O. Kaplan, S. Kloster, W. Knorr, G. Lasslop, F. Li, S. Mangeon, J. R. Melton, A. Meyn, S. Sitch, A. Spessa, G. R. van der Werf, A. Voulgarakis, C. Yue, The status and challenge of global fire modelling. Biogeosciences 13, 3359–3375 (2016).

[64] P. Nyman, C. C. Baillie, T. J. Duff, G. J. Sheridan, Eco-hydrological controls on microclimate and surface fuel evaporation in complex terrain. Agric. For. Meteorol. 252, 49–61 (2018).

[65] T. P. Brown, A. Inbar, T. J. Duff, J. Burton, P. J. Noske, P. N. J. Lane, G. J. Sheridan, Forest structure drives fuel moisture response across alternative forest states. Fire 4, 48 (2021).

[66] X. Liu, P.-L. Ma, H. Wang, S. Tilmes, B. Singh, R. C. Easter, S. J. Ghan, P. J. Rasch, Description and evaluation of a new four-mode version of the Modal Aerosol Module (MAM4) within version 5.3 of the Community Atmosphere Model. Geosci. Model Dev. 9, 505–522 (2016).

[67] X. Liu, R. C. Easter, S. J. Ghan, R. Zaveri, P. Rasch, X. Shi, J.-F. Lamarque, A. Gettelman, H. Morrison, F. Vitt, A. Conley, S. Park, R. Neale, C. Hannay, A. M. L. Ekman, P. Hess, N. Mahowald, W. Collins, M. J. Iacono, C. S. Bretherton, M. G. Flanner, D. Mitchell, Toward a minimal representation of aerosols in climate models: Description and evaluation in the Community Atmosphere Model CAM5. Geosci. Model Dev. 5, 709–739 (2012).

[68] S. Tilmes, M. J. Mills, Y. Zhu, C. G. Bardeen, F. Vitt, P. Yu, D. Fillmore, X. Liu, B. Toon, T. Deshler, Description and performance of a sectional aerosol microphysical model in the Community Earth System Model (CESM2). Geosci. Model Dev. 16, 6087–6125 (2023).

[69] F. Li, X. D. Zeng, S. Levis, Corrigendum to “A process-based fire parameterization of intermediate complexity in a Dynamic Global Vegetation Model”. Biogeosciences 9, 4771–4772 (2012).

[70] F. Li, S. Levis, D. S. Ward, Quantifying the role of fire in the Earth system Part 1: Improved global fire modeling in the Community Earth System Model (CESM1). Biogeosciences 10, 2293–2314 (2013).

[71] F. Li, D. M. Lawrence, B. Bond-Lamberty, Impact of fire on global land surface air temperature and energy budget for the 20th century due to changes within ecosystems. Environ. Res. Lett. 12, 044014 (2017).

[72] F. Li, D. M. Lawrence, Role of fire in the global land water budget during the twentieth century due to changing ecosystems. J. Clim. 30, 1893–1908 (2017).

[73] F. Li, D. M. Lawrence, B. Bond-Lamberty, Human impacts on 20th century fire dynamics and implications for global carbon and water trajectories. Glob. Planet. Change 162, 18–27 (2018).

[74] C. Wiedinmyer, Y. Kimura, E. C. McDonald-Buller, L. K. Emmons, R. R. Buchholz, W. Tang, K. Seto, M. B. Joseph, K. C. Barsanti, A. G. Carlton, R. Yokelson, The Fire Inventory from NCAR version 2.5: An updated global fire emissions model for climate and chemistry applications. Geosci. Model Dev. 16, 3873–3891 (2023).

[75] J. Randerson, G. van der Werf, L. Giglio, G. Collatz, P. Kasibhatla, Global Fire Emissions Database, Version 4.1 (GFEDv4) (ORNL DAAC, 2018).

[76] P. DeRepentigny, A. Jahn, M. M. Holland, J. E. Kay, J. Fasullo, J.-F. Lamarque, S. Tilmes, C. Hannay, M. J. Mills, D. A. Bailey, A. P. Barrett, Enhanced simulated early 21st century Arctic sea ice loss due to CMIP6 biomass burning emissions. Sci. Adv. 8, eabo2405 (2022). 35895816 10.1126/sciadv.abo2405 PMC9328692

[77] J. T. Fasullo, J.-F. Lamarque, C. Hannay, N. Rosenbloom, S. Tilmes, P. DeRepentigny, A. Jahn, C. Deser, Spurious Late Historical-Era Warming in CESM2 Driven by prescribed biomass burning emissions. Geophys. Res. Lett. 49, e2021GL097420 (2022).

[78] G. D. Thornhill, W. J. Collins, R. J. Kramer, D. Olivié, R. B. Skeie, F. M. O’Connor, N. L. Abraham, R. Checa-Garcia, S. E. Bauer, M. Deushi, L. K. Emmons, P. M. Forster, L. W. Horowitz, B. Johnson, J. Keeble, J.-F. Lamarque, M. Michou, M. J. Mills, J. P. Mulcahy, G. Myhre, P. Nabat, V. Naik, N. Oshima, M. Schulz, C. J. Smith, T. Takemura, S. Tilmes, T. Wu, G. Zeng, J. Zhang, Effective radiative forcing from emissions of reactive gases and aerosols–a multi-model comparison. Atmos. Chem. Phys. 21, 853–874 (2021).

