# Supplementary material for: Are Northern Hemisphere boreal forest fires more sensitive to future aerosol mitigation than to greenhouse gas–driven warming?
Source: Sci Adv. 2024 Mar 29;10(13):eadl4007. doi: 10.1126/sciadv.adl4007 (PMC10980282; doi:10.1126/sciadv.adl4007)
Supplement: Supplementary file 1 — Notes S1 to S6 Figs. S1 to S17 [file sciadv.adl4007_sm.pdf]

Science Advances
AAAS

Supplementary Materials for

Are Northern Hemisphere boreal forest fires more sensitive to future aerosol mitigation than to greenhouse gas–driven warming?

Robert J. Allen *et al.*

Corresponding author: Robert J. Allen, rjallen@ucr.edu

Sci. Adv. **10**, eadl4007 (2024)
DOI: 10.1126/sciadv.adl4007

This PDF file includes:

Notes S1 to S6
Figs. S1 to S17

Supplementary Note 1: AOD Trends and Warming

Supplementary Figure 1 shows 2015-2060 zonal mean annual mean land-only aerosol optical depth (AOD) trends. Supplementary Figure 1a-c (top panels) shows AOD trends for the base (ssp370; black) and perturbation (ssp370-126aer; gray) experiments and Supplementary Figure 1d-f (bottom panels) shows their difference, i.e., aerosol mitigation (red lines). As expected, aerosol mitigation leads to robust reductions in aerosol optical depth (AOD), with maximum reductions near 20°N (Supplementary Fig. 1d). Over global land, AOD decreases by −16.2% relative to the 2000-2014 climatology. Similarly, sulfate (SO_4) AOD decreases by −57.7% (e.g., Supplementary Fig. 1e) and black carbon (BC) AOD decreases by −78.1% (e.g., Supplementary Fig. 1f). Other anthropogenic aerosol species including primary and secondary organic aerosol also exhibit large decreases under aerosol mitigation (Supplementary Figure 2).

The AOD decrease under aerosol mitigation is due to relatively large AOD decreases under ssp370-126aer (consistent with the strong air quality control measures in SSP1-2.6) and small AOD changes under ssp370 (consistent with weak air quality control measures). In particular, negligible changes in AOD occur in ssp370 over the NH extratropics (30-90°N; Supplementary Fig. 1a-c). Supplementary Figure 3a shows a corresponding non-significant 0.9% AOD increase. Similar conclusions apply for the NH boreal forest region, where ssp370 AOD increases by a non-significant 2.6%. Thus, aerosol contributions to ssp370 NH extratropical climate change, including the boreal forest region, are minimal (i.e., ssp370 climate change in this region is largely due to the increase in GHGs).

The increase in GHGs under ssp370 and the AOD reduction under aerosol mitigation leads to significant warming (Supplementary Figure 4). For example, over the NH boreal for-

est region, ssp370 yields warming (largely due to increasing GHGs) of 0.56±0.04 K decade^{-1}. Larger warming, due to both increasing GHGs and aerosol reductions, occurs under ssp370-126aer at 0.89±0.02 K decade^{-1}. Thus, aerosol mitigation (ssp370-126aer−ssp370) yields significant warming at 0.33±0.04 K decade^{-1}, about 60% as large as that under ssp370.

Supplementary Note 2: Additional Fire Drivers

Although aerosol mitigation features multi-model mean decreases in surface wind speed and increases in precipitation over the boreal forest region (which would promote decreases in fire activity; Supplementary Figure 7), we also compare their trends with FIREC trends. For near-surface wind speed, non-significant correlations occur in all cases, including both positive and negative correlations. For example, the aerosol mitigation FIREC versus surface wind speed trend correlation over the boreal forest region is −0.20 and 0.08 for the annual and JJA mean, respectively. The corresponding FIREC versus precipitation trend correlations over the boreal forest region are both negative (as expected) at −0.38 for ANN and −0.54 for JJA, but these are not significant at the 90% confidence level. Moreover, this result implies that aerosol mitigation realizations with a larger increase in precipitation (all 10 realizations feature an increase in precipitation) are associated with a weaker increase in FIREC (again, all 10 realizations feature an increase in FIREC)−this does not help us understand FIREC amplification under aerosol mitigation.

Although human fire ignition/suppression effects on wildfire activity are included in the CLM fire module (parameterized as a function of both population density and gross domestic product), such effects are likely minimal in the boreal region, due to limited human population in this region. And more importantly, such effects are minimal due to small

changes (weak decreases) in future projected population in the boreal region under SSP3-7.0 (Supplementary Figure 16). Note that human effects on ignition and suppression do not impact aerosol mitigation (since it is estimated from the difference of two experiments with identical changes in population, etc.).

Similarly, although our CESM2 simulations feature changes in land use/land change from the Shared Socioeconomic Pathway 3-7.0 (SSP3-7.0), changes in land use/land change will not impact aerosol mitigation. This is because aerosol mitigation is estimated from the difference of two experiments with identical changes in land use/land change. Land use/land change also likely has minimal impacts on FIREC trends in ssp370, as the NH boreal forest region is not a region subject to significant anthropogenic land use/land change disturbances. For example, the 2015-2060 trends in crop, tree and grass fraction in the NH boreal forest region are all relatively small and not significant (Supplementary Figure 17).

Nonetheless, we acknowledge that we cannot completely rule out potential impacts of population and land use change on FIREC trends in the baseline ssp370 experiment. The only way to do this cleanly would be to conduct another 10 ensemble member set of CESM2 simulations that are identical to ssp370, but feature GHG concentrations fixed to their 2015 values (subtracting with ssp370 will yield the pure GHG signal without possible influences of population and land use change).

Supplementary Note 3: Plant Physiological Responses to CO_2

The weaker latent heat flux and canopy transpiration increase under ssp370 (relative to aerosol mitigation) is consistent with plant physiological responses to CO_2, which includes reduced stomatal conductance and water use. For example, JJA stomatal conductance for

sunlit and shaded leaves under ssp370 decreases by -6.3 ± 0.1 and -3.0 ± 0.03 μmol H_20 m^{-2} s^{-1} $decade^{-1}$ (Supplementary Figure 12g,h). The corresponding percent changes (relative to the 2000-2014 climatology) are quite large at −30.9% and −38.1%, respectively. The stomatal conductance trends under aerosol mitigation, however, are much smaller (and negative for stomatal conductance for shaded leaves) at 0.21 ± 0.10 and -0.27 ± 0.06 μmol H_20 m^{-2} s^{-1} $decade^{-1}$. We also note that the large ssp370 increase in SW_{in} and decrease in cloud cover during JJA is to some extent likely related to the biogeophysical effects of CO_2 on vegetation, in addition to other GHG-induced cloud feedbacks. The (relatively weak) increase in canopy transpiration under ssp370, despite a decrease in stomatal conductance, is likely related to the large increase in vegetation indices, including net primary productivity (NPP; Supplementary Figure 12e) and leaf area index (LAI; Supplementary Figure 12f). For example, leaf area index (LAI) increases by 0.17 ± 0.009 $decade^{-1}$, or 26.1%, in JJA under ssp370.

Supplementary Note 4: Surface Water Balance

Changes in surface water are related to precipitation minus evapotranspiration. Additional terms of the surface water balance include snowmelt (an input) and runoff (an output). However, these terms are not responsible for amplification of soil drying in the boreal forest region under aerosol mitigation (Supplementary Figure 14). For example, precipitation increases in all seasons under aerosol mitigation (Supplementary Fig. 14a). The relatively large increase in evapotranspiration offsets (Supplementary Fig. 14b) much of this precipitation increase in most seasons, including JJA, leading to a negative precipitation minus evapotranspiration trend (Supplementary Fig. 14e) of -0.019 ± 0.011 mm day^{-1} $decade^{-1}$. Due to

the decrease in ssp370 precipitation during JJA, ssp370 actually yields a larger negative precipitation minus evapotranspiration trend at -0.040 ± 0.08 mm day^{-1} decade^{-1}. Snowmelt significantly decreases, largely in MAM, due to less snow fall (more falls as rain) and less snow on the ground. Although this acts to dry the soil, the changes are very similar between aerosol mitigation and ssp370 (Supplementary Fig. 14c). Relatively small changes in surface runoff occurs, with aerosol mitigation showing negligible trend in JJA. The combined effect (precipitation minus evapotranspiration plus snow melt minus runoff; Supplementary Fig. 14f) yields significant decreasing trends for MAM and JJA, but with similar values between ssp370 and aerosol mitigation. For example, during JJA, the trends are -0.022 ± 0.009 and -0.034 ± 0.006 mm day^{-1} decade^{-1} for aerosol mitigation and ssp370. The trends are actually slightly more negative for ssp370, implying more drying for ssp370 as opposed to aerosol mitigation.

Supplementary Note 5: Additional Analysis of Soil Liquid/Ice Trends

The negative trend correlations between soil ice and soil liquid do not occur during JJA or SON (Supplementary Figure 15a,b). Interpretation during these seasons is more difficult (especially closer to the surface), as JJA/SON soil liquid trends are not only influenced by melting of soil ice (acting to increase soil liquid), but also by evapotranspiration (acting to decrease soil liquid). This is reflected by the positive JJA trend correlations between soil ice and soil liquid from the surface down to $\sim$1 m depth (Supplementary Figure 15a,b). These positive trend correlations indicate decreases in both soil ice and soil liquid here (along with Figure 6 from the main paper), suggesting the increase in summertime evapotranspiration (which acts to reduce soil liquid water) dominates over conversion of soil ice to soil liquid.

Supplementary Figure 15c,d shows seasonally lagged soil liquid trend correlations. Significant positive correlations at depth (from ∼0.5 to 3 m) exist between MAM and the previous season (DJF) for both ssp370 and aerosol mitigation. However, only ssp370 yields significant positive correlations at depth (from ∼1.5 to 3 m) between JJA soil liquid and the previous season (MAM). Thus, this suggests the increase in JJA soil liquid at depth under ssp370 is related to carry-over of melted MAM soil ice.

We also note the positive lagged correlations (particularly for aerosol mitigation; Supplementary Figure 15d) near the surface down to ∼1m depth between SON and the previous season, JJA, suggesting SON soil drying near the surface is related to JJA drying (i.e., realizations with larger SON decreases in soil liquid are associated with larger decreases in JJA soil liquid).

The negative JJA lagged correlations near the surface (Supplementary Figure 15c,d) suggest larger JJA drying is associated with larger increases in MAM soil liquid. This also implies a carry-over of moisture−with more soil liquid in MAM near the surface, there is more to be evapotranspirated in the subsequent season.

Supplementary Note 6: Depth of RZSW Impacts on Soil Liquid Water

Here, we infer the depths to which RZSW impacts soil liquid water. The largest trend correlations (over realizations) between RZSW and soil liquid water during JJA over the boreal forest region occur at depths between 0.80 and 1.36 m. For example, the trend correlations between RZSW and soil liquid water at 1.06 m depth are 0.74 and 0.73 for aerosol mitigation and ssp370, respectively. This is also consistent with the plant function type root distribution parameters, as 90% of the roots are located within ∼1.2 m of the surface for

most tree species (as well as grasses and shrubs). This includes boreal tree species, such as needleleaf deciduous, needleleaf evergreen and broadleaf deciduous. Thus, over the boreal forest region, RZSW captures soil liquid water changes down to $\sim$1 m depth.

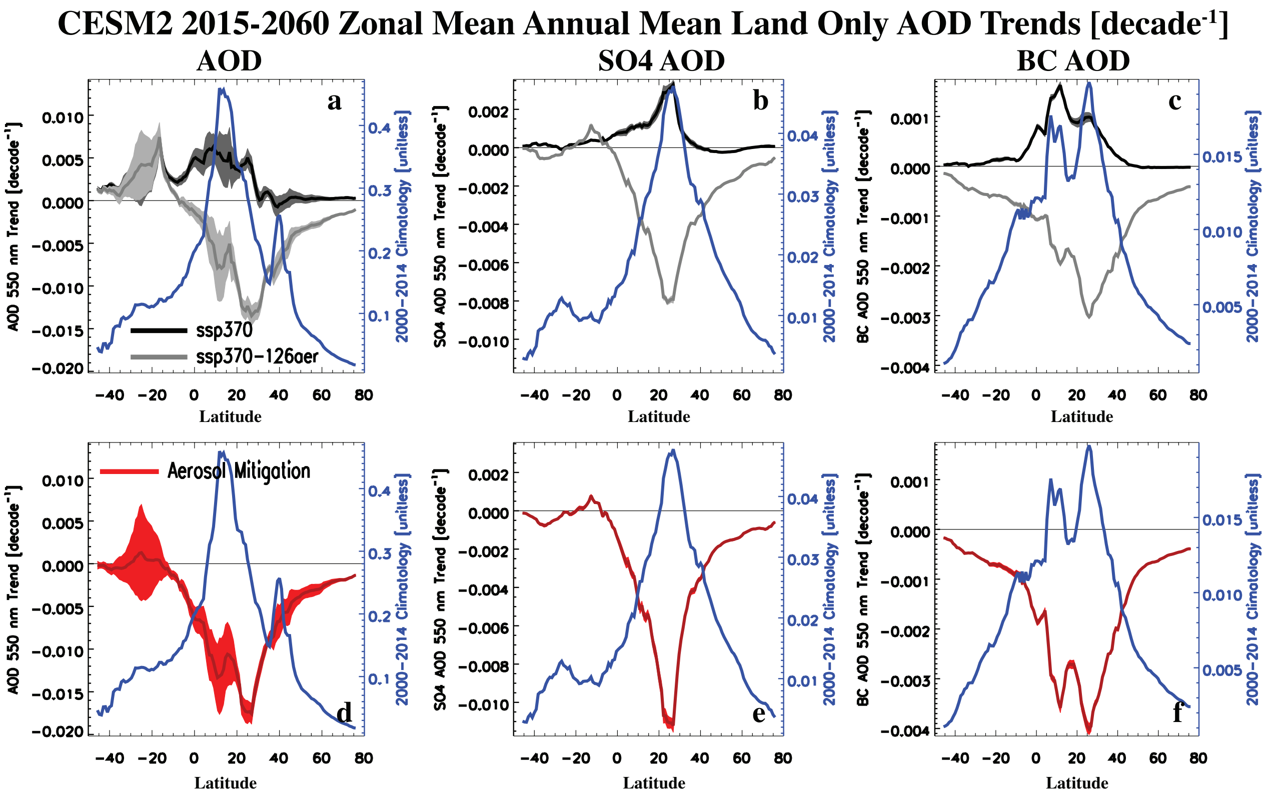

Supplementary Figure 1 **Zonal mean aerosol optical depth trends.** 2015-2060 zonal mean annual mean land-only aerosol optical depth (AOD) trends and 2000-2014 climatologies (blue; right y-axis) for (a, d) AOD 550 nm; (b, e) sulfate AOD 550 nm; and (c, f) black carbon AOD 550 nm. Top panels (a-c) show trends from the perturbation (ssp370-126aer; gray) and base (ssp370; black) experiments; bottom panels (d-f) shows the aerosol mitigation signal (i.e., ssp370-126aer−ssp370; red). Trend units are decade^{-1} and climatology units are dimensionless. Shading shows the 1-standard deviation uncertainty across the 10 ensemble members. Throughout this entire manuscript (Methods), trends are estimated from a standard least-squares regression.

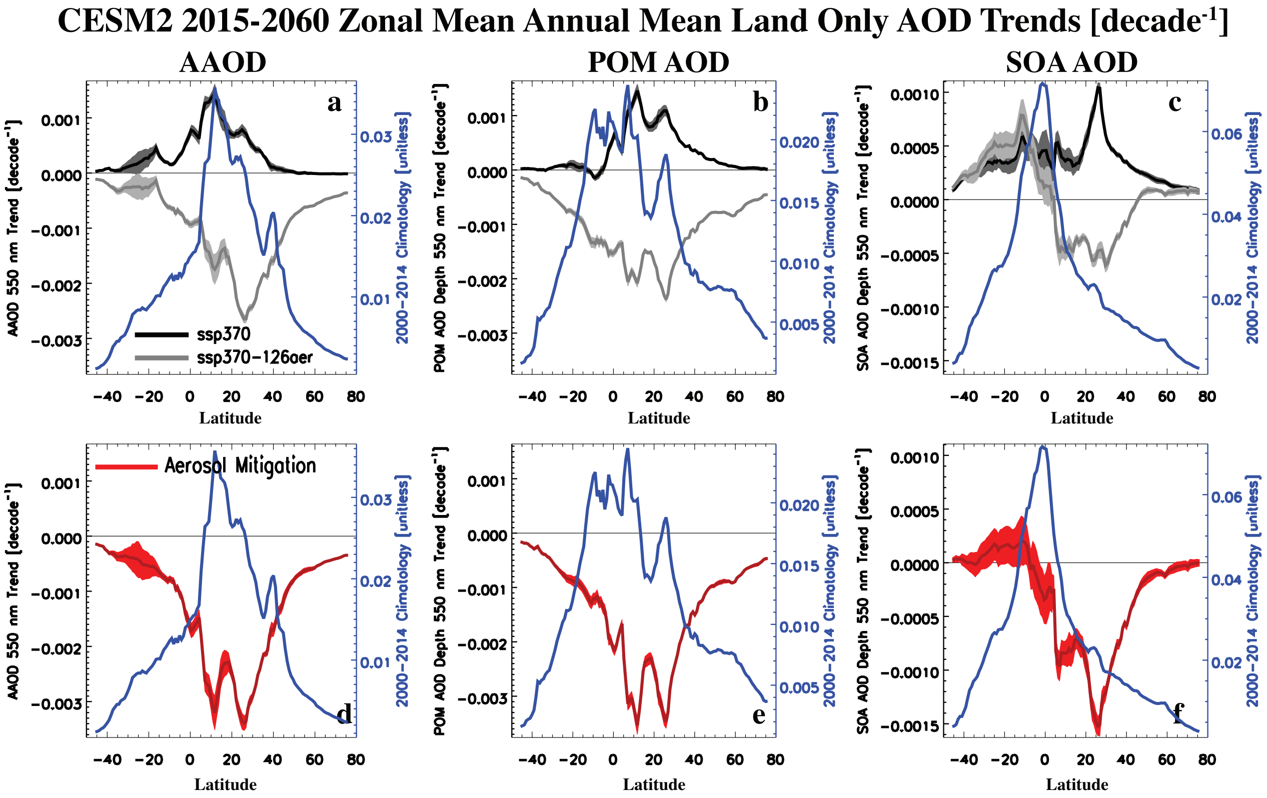

Supplementary Figure 2 **Zonal mean aerosol optical depth trends.** 2015-2060 zonal mean annual mean land only aerosol optical depth (AOD) trends and 2000-2014 climatologies (blue; right y-axis) for (a, d) absorption AOD 550 nm; (b, e) primary organic matter AOD 550 nm; and (c, f) secondary organic aerosol AOD 550 nm. Top panels (a-c) show trends from the perturbation (ssp370-126aer; gray) and base (ssp370; black) experiments; bottom panels (d-f) shows the aerosol mitigation signal (i.e., ssp370-126aer−ssp370; red). Trend units are decade^{-1} and climatology units are dimensionless. Shading shows the 1-standard deviation uncertainty across the 10 ensemble members. Absorption AOD decreases by −46.4%; primary organic matter AOD decreases by −50.5%; and secondary organic aerosol AOD decreases by −4.7% (relative to the 2000-2014 climatology).

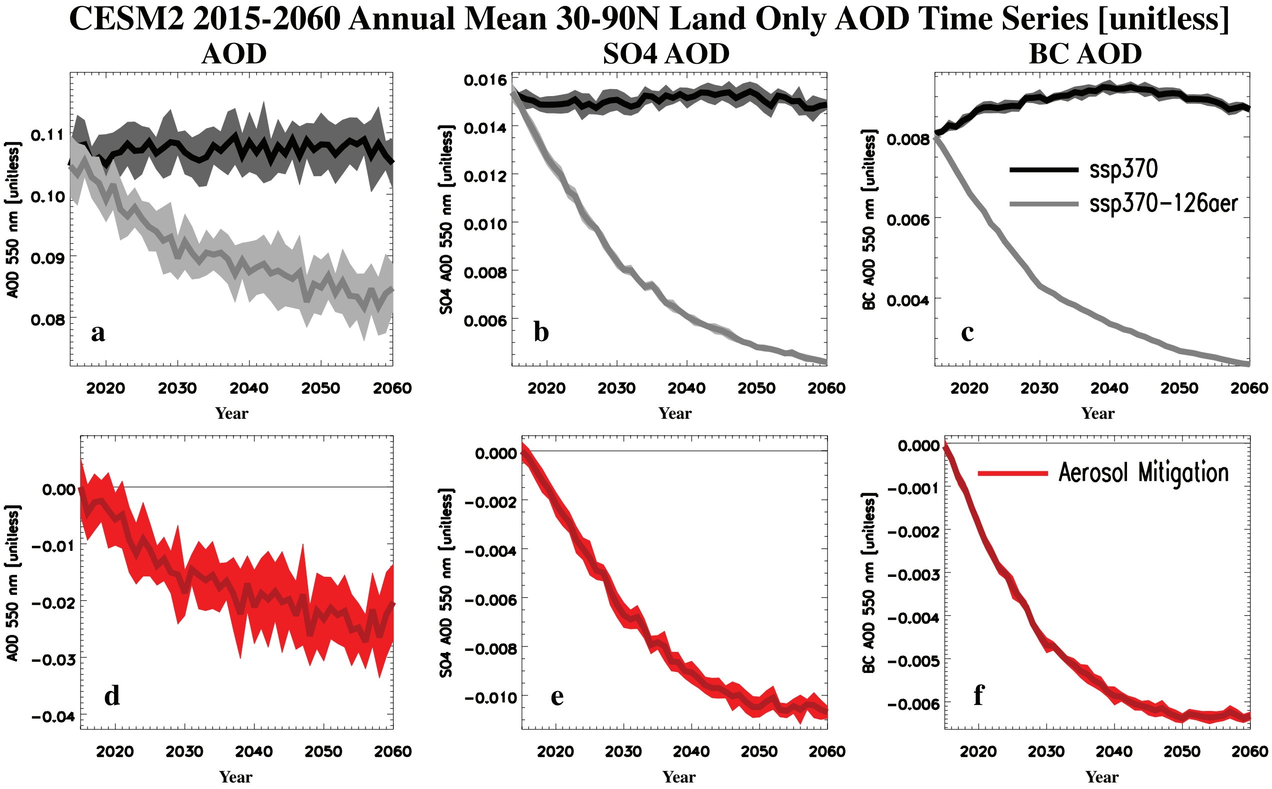

Supplementary Figure 3 **Aerosol optical depth time series.** 2015-2060 annual mean NH extratropical (30-90°N) land only aerosol optical depth (AOD) time series for (a, d) AOD 550 nm; (b, e) sulfate AOD 550 nm; and (c, f) black carbon AOD 550 nm. Top panels (a-c) show time series from the perturbation (ssp370-126aer; gray) and base (ssp370; black) experiments; bottom panels (d-f) shows the aerosol mitigation time series (i.e., ssp370-126aer−ssp370; red). AOD units are dimensionless. Shading shows the 1-standard deviation uncertainty across the 10 ensemble members.

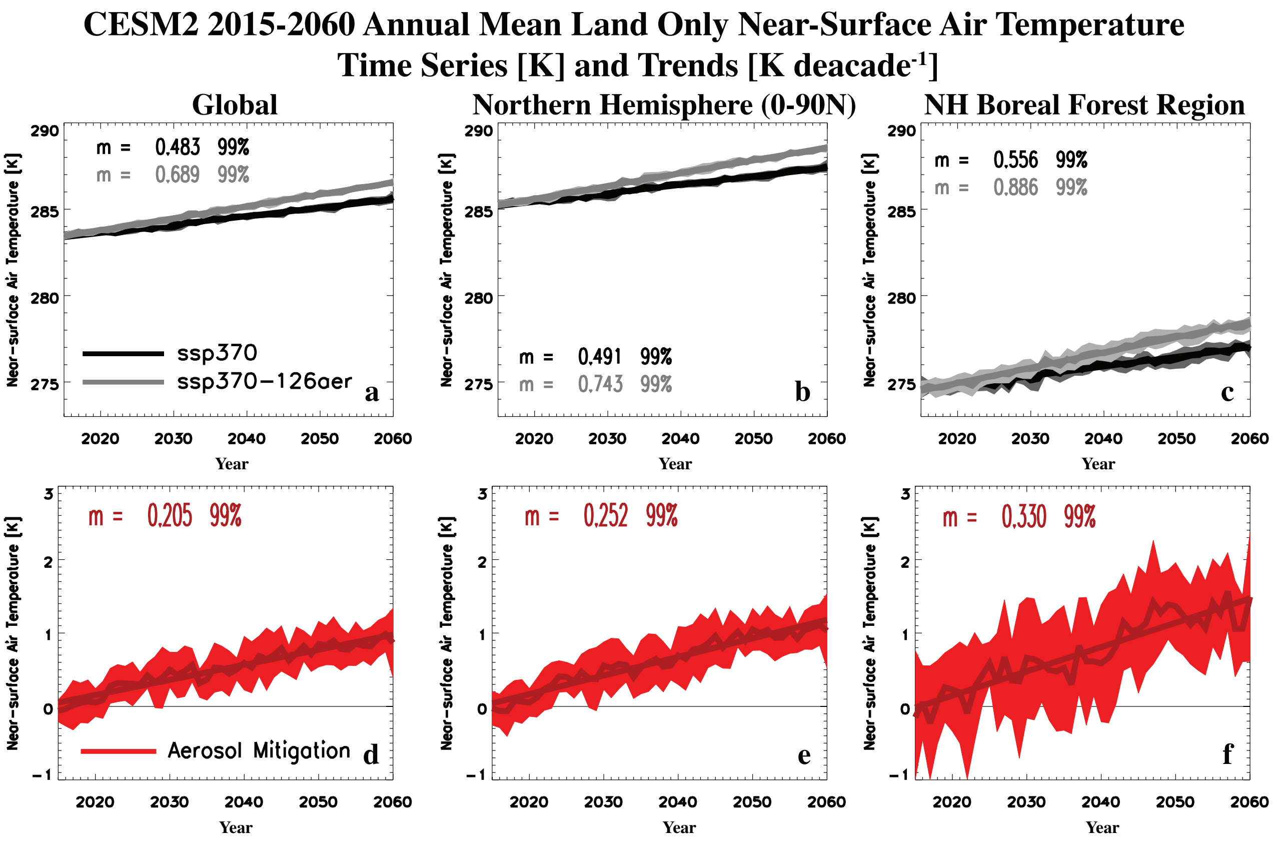

Supplementary Figure 4 **Near-surface air temperature time series.** 2015-2060 annual mean land only near-surface air temperature (TAS) time series for the (a, d) global mean; (b, e) Northern Hemisphere (0-90°N) mean; and (c, f) Northern Hemisphere boreal forest region (45-90°N with tree fraction >50%) mean. Top panels (a-c) show time series from the perturbation (ssp370-126aer; gray) and base (ssp370; black) experiments; bottom panels (d-f) shows the aerosol mitigation time series (i.e., ssp370-126aer−ssp370; red). TAS units are K and trend units are K decade^{-1}. Shading shows the 1-standard deviation uncertainty across the 10 ensemble members. Also included is the slope of the 2015-2060 least squares trend line [K decade^{-1}] and its significance based on a standard two-tailed t-test.

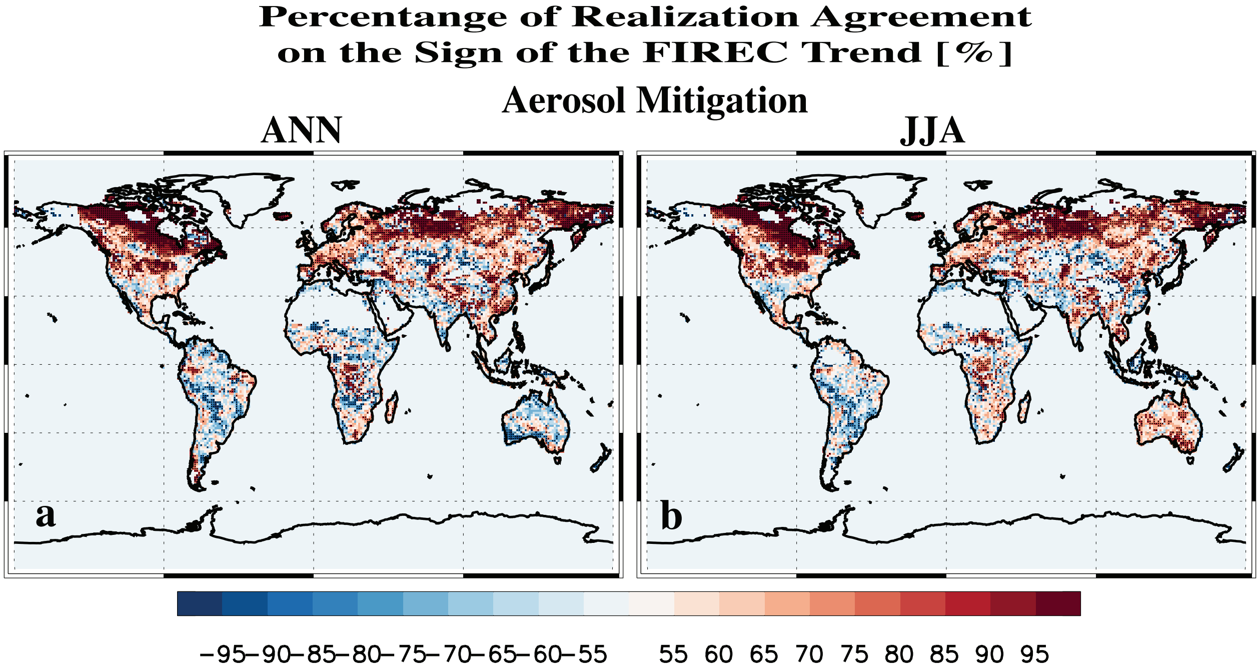

Supplementary Figure 5 **Percentage of realization agreement of the sign of the FIREC trend under aerosol mitigation.** (a) Annual and (b) June-July-August (JJA) mean realization agreement of the sign of the fire carbon emissions trend. Units are %. Dots represent a significant response at the 90% confidence level based on a binomial test. To illustrate what is being plotted, an 80% trend realization agreement means 8 of the 10 ensemble members simulate a positive FIREC trend in that grid box. Similarly, a −80% trend realization agreement means 8 of the 10 ensemble members simulate a negative FIREC trend in that grid box.

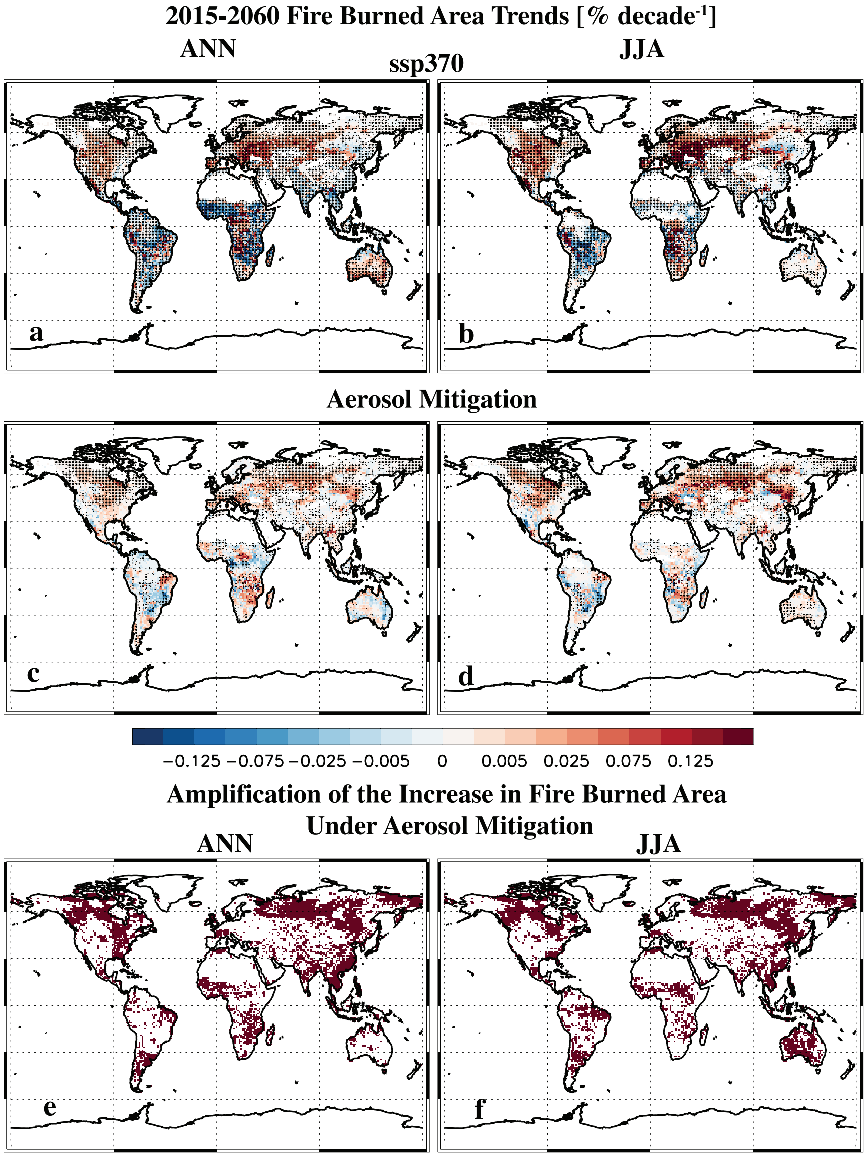

Supplementary Figure 6 **2015-2060 fire burned area trend maps.** (a, c) Annual mean and (b, d) June-July-August (JJA) mean fire burned area trends for (a, b) ssp370 and (c, d) aerosol mitigation (i.e., ssp370-126aer−ssp370). Trend units are % decade^{-1}. Dots represent a significant response at the 90% confidence level based on a standard two-tailed t-test. Also included are (e) annual and (f) JJA maps showing amplification of the increase in fire burned area under aerosol mitigation (relative to ssp370). Red shading denotes regions where aerosol mitigation yields larger increases relative to ssp370 (i.e., percent change exceeds 100%) or aerosol mitigation yields positive trends whereas ssp370 yields negative trends.

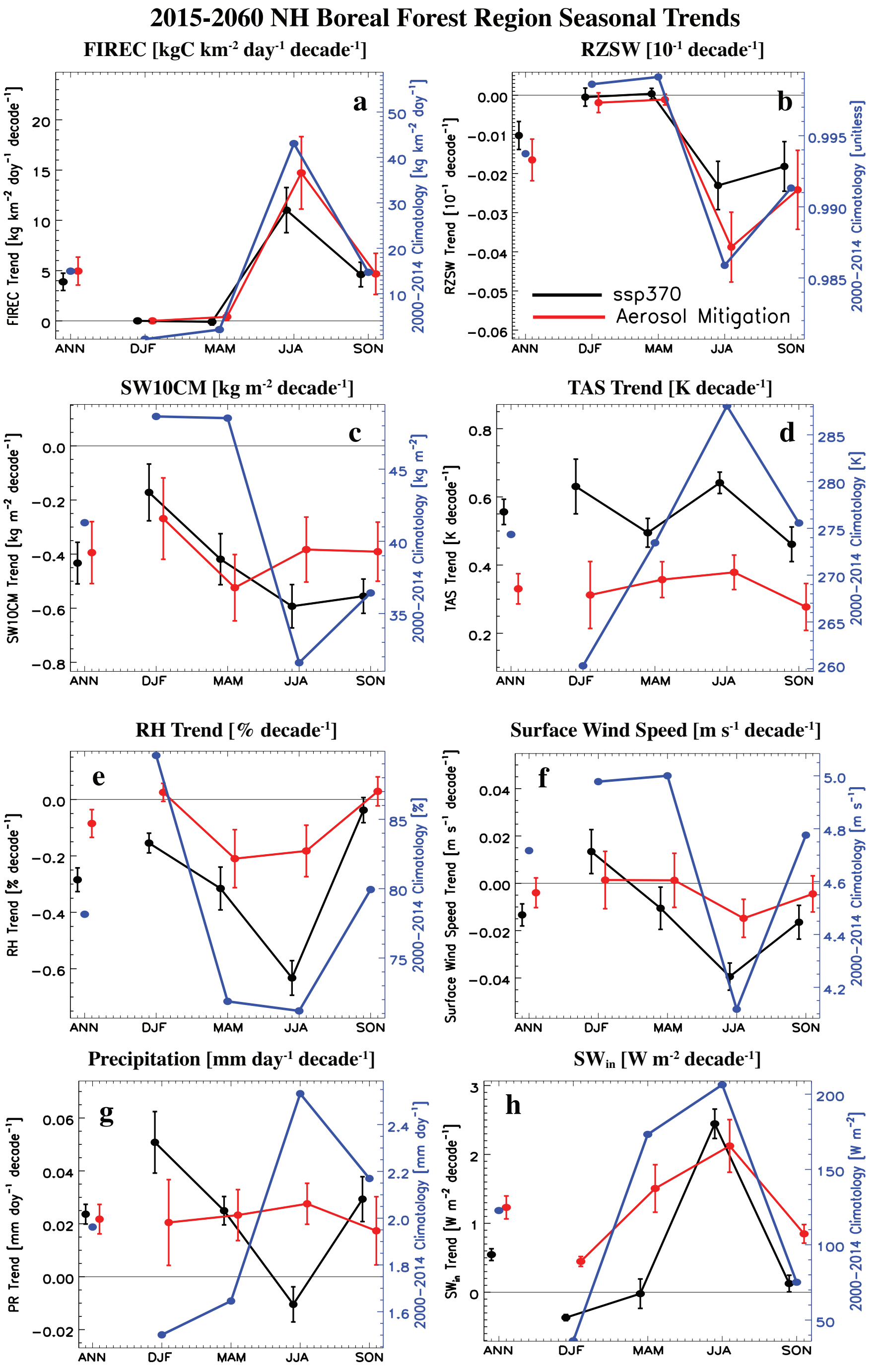

Supplementary Figure 7 **2015-2060 NH boreal forest region seasonal trends.** NH boreal forest region seasonal trends for (a) fire carbon emissions (FIREC; [kgC km^{-2} day^{-1} decade^{-1}]); (b) root-zone soil wetness (RZSW; [10^{-1} decade^{-1}]); (c) soil water upper 10 cm (SW10CM; [kg m^{-2} decade^{-1}]); (d) near-surface air temperature (TAS; [K decade^{-1}]); (e) near-surface relative humidity (RH; [% decade^{-1}]); (f) surface wind speed ([m s^{-1} decade^{-1}]); (g) precipitation [mm day^{-1} decade^{-1}]; and (h) surface downwelling shortwave radiation (SW_{in}; [W m^{-2} decade^{-1}]). In addition to 2015-2060 ssp370 (black) and aerosol mitigation (red) trends, the 2000-2014 climatology (blue) is also included. Seasons include December-January-February (DJF), March-April-May (MAM), June-July-August (JJA) and September-October-November (SON). Also included is the annual mean (ANN). Error bars show the 90% confidence interval of the trend, estimated as $\frac{1.65\times\sigma}{\sqrt{n-1}}$, where σ is the standard deviation across the trends and n is the number of trends (i.e., 10). Panels a (FIREC); b (RZSW); and h (SW_{in}) are identical to figures from the main paper.

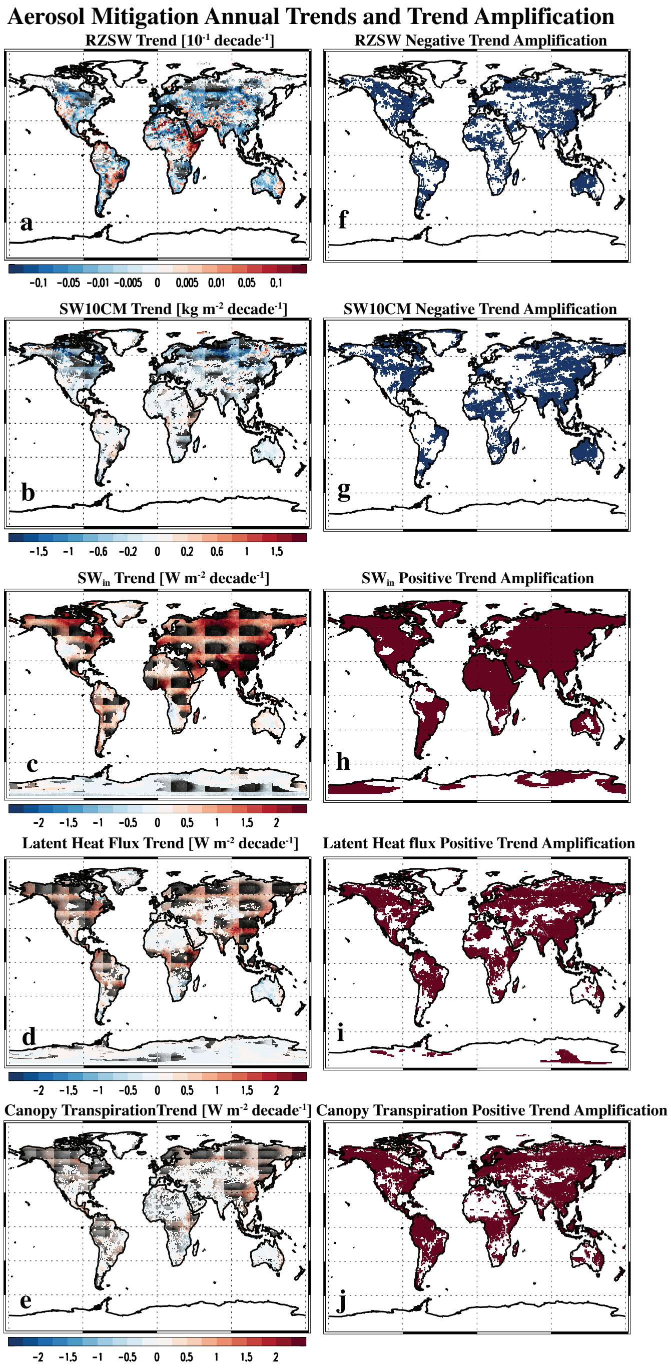

SUPPLEMENTARY FIGURE 8 **2015-2060 aerosol mitigation trend and trend amplification maps.** (a-e) Annual mean trends and (f-j) trend amplification under aerosol mitigation (relative to ssp370). Annual mean trends of (a) root-zone soil wetness (RZSW; [10^{-1} decade^{-1}]); (b) soil water upper 10 cm (SW10CM; [kg m^{-2} decade^{-1}]; (c) surface downwelling shortwave radiation (SW_{in}; [W m^{-2} decade^{-1}]); (d) surface latent heat flux [W m^{-2} decade^{-1}] and (e) canopy transpiration [W m^{-2} decade^{-1}]. Dots represent a significant response at the 90% confidence level based on a standard two-tailed t-test. Trend amplification under aerosol mitigation for (f) RZSW; (g) SW10CM; (h) SW_{in}; (i) latent heat flux and (j) canopy transpiration. Any shaded region (whether red or blue) shows amplification of the trend under aerosol mitigation (amplification of the positive trend in SW_{in}, latent heat flux and canopy transpiration; amplification of the negative trend in RZSW and SW10CM). Panels a and f (RZSW) are identical to Figure 2 from the main paper.

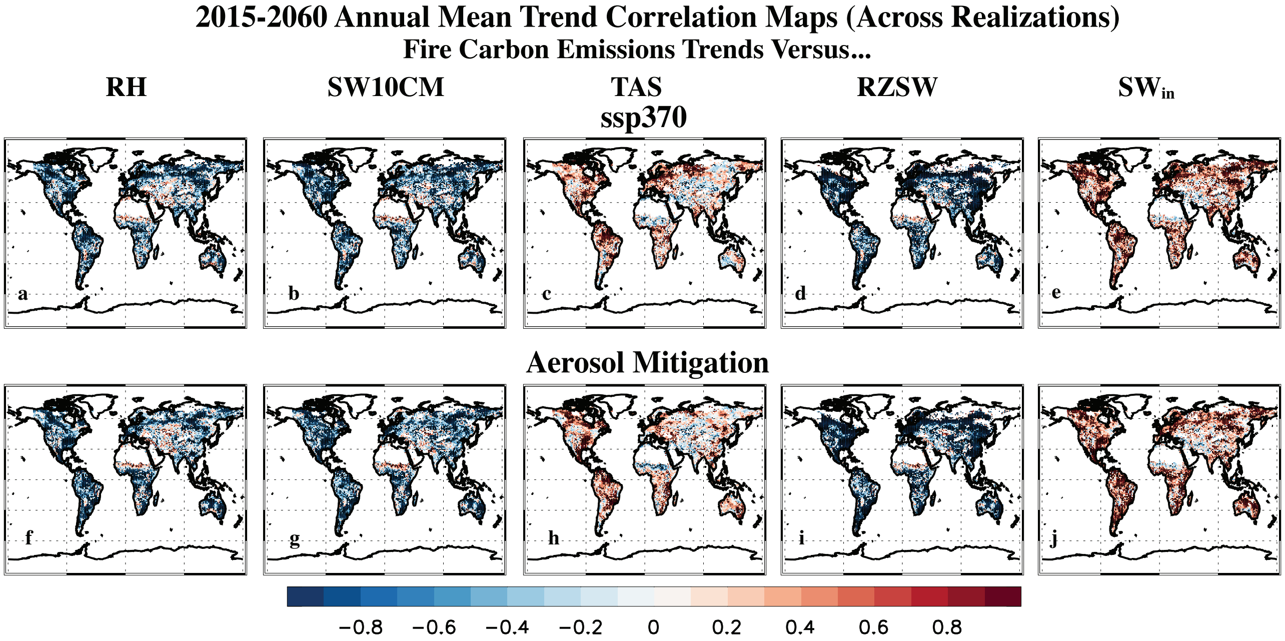

Supplementary Figure 9 **2015-2060 trend correlation maps.** 2015-2060 correlations between annual mean fire carbon emissions (FIREC) trends and (a, f) near-surface relative humidity (RH); (b, g) soil water upper 10 cm (SW10CM); (c, h) near-surface air temperature (TAS); (d, i) root-zone soil wetness (RZSW); and (e, j) surface downwelling shortwave radiation (SW_{in}) trends for (a-e) ssp370 and (g-j) aerosol mitigation. Correlations are over the trends of the ten ensemble members at each grid box. Dots represent a correlation significant at the 90% confidence level, based on a standard t-test.

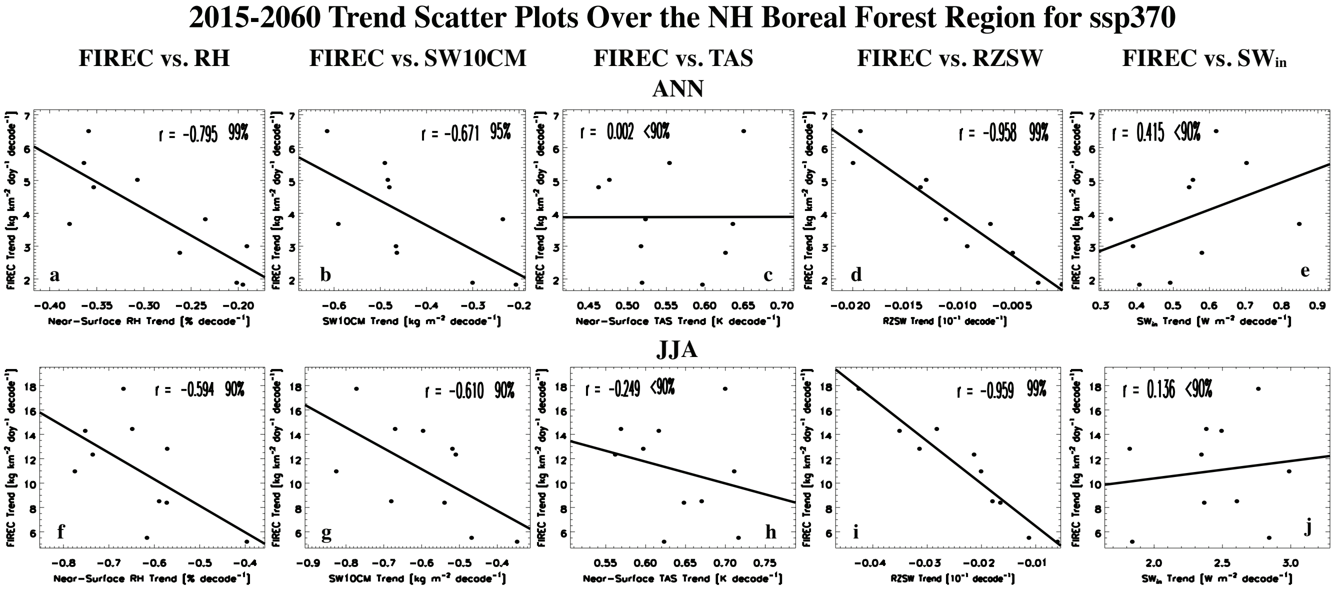

Supplementary Figure 10 **2015-2060 ssp370 trend scatter plots.** 2015-2060 NH boreal forest region fire carbon emissions (FIREC) trends [kgC km^{-2} day^{-1} decade^{-1}] versus corresponding trends in (a, f) near-surface relative humidity (RH; [% decade^{-1}]); (b, g) soil water upper 10 cm (SW10CM; [kg m^{-2} decade^{-1}]); (c, h) near-surface air temperature (TAS; [K decade^{-1}]); (d, i) root-zone soil wetness (RZSW; [10^{-1} decade^{-1}]); and (e, j) surface downwelling shortwave radiation (SW_{in}) for the (a-e) annual mean and (f-j) JJA mean. Each dot represents the trend from one of the ten ensemble members under ssp370. Also included is the correlation coefficient, r, and its significance based on a standard t-test.

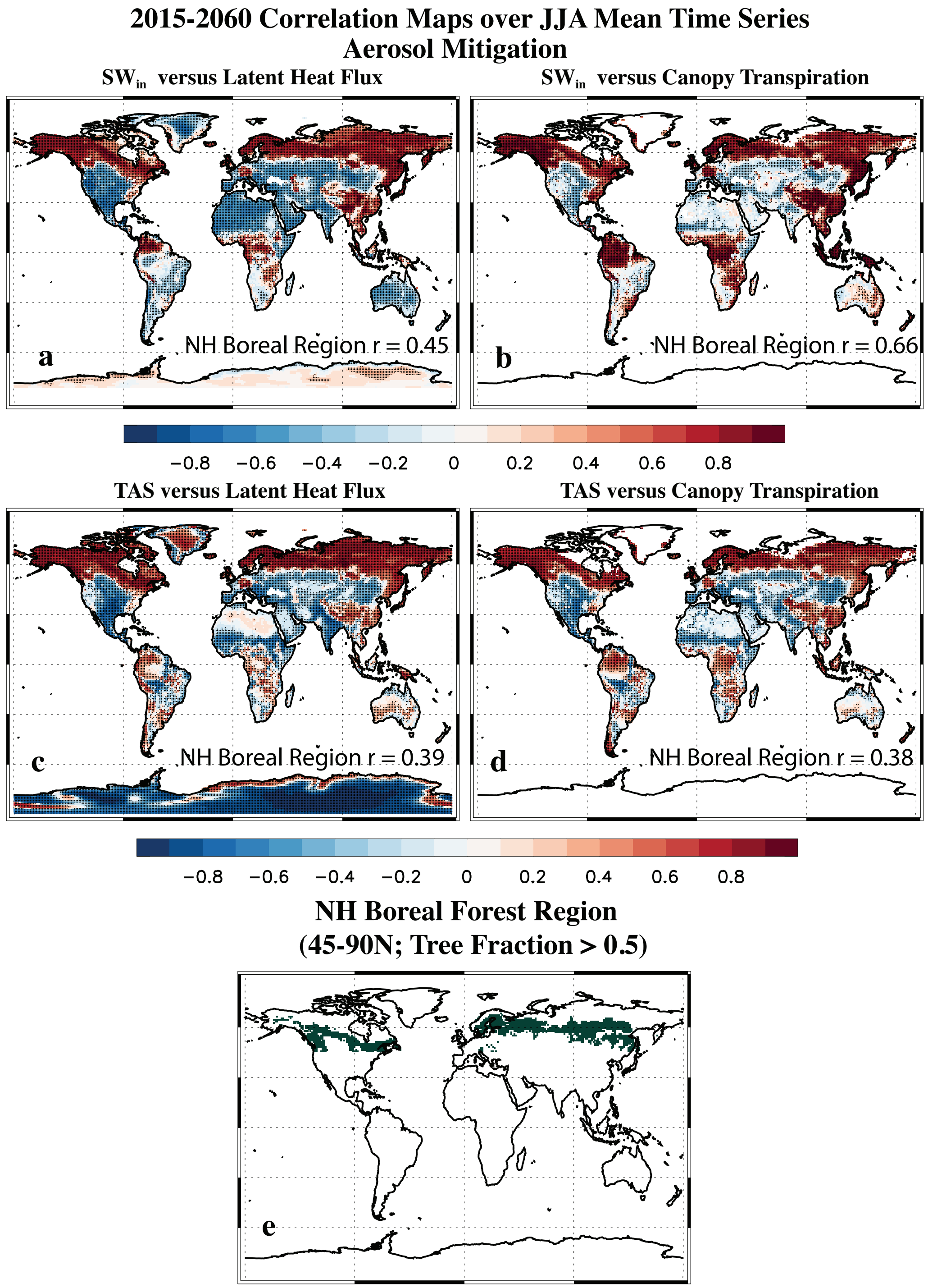

Supplementary Figure 11 **2015-2060 aerosol mitigation correlation maps.** Grid box correlations based on JJA mean time series between downwelling surface shortwave radiation (SW_{in}) and (a) surface latent heat flux and (b) canopy transpiration. Corresponding correlations between near-surface air temperature (TAS) and (c) latent heat flux and (d) canopy transpiration. Dots represent a significant correlation at the 90% confidence level based on a standard t-test. Panel (e) shows the NH boreal forest region (i.e., dark green shading) as defined here, as grid boxes over land from 45-90°N with at least 50% tree fraction. NH boreal forest region average correlations are included in each panel, e.g., r = 0.45 for SW_{in} versus latent heat flux.

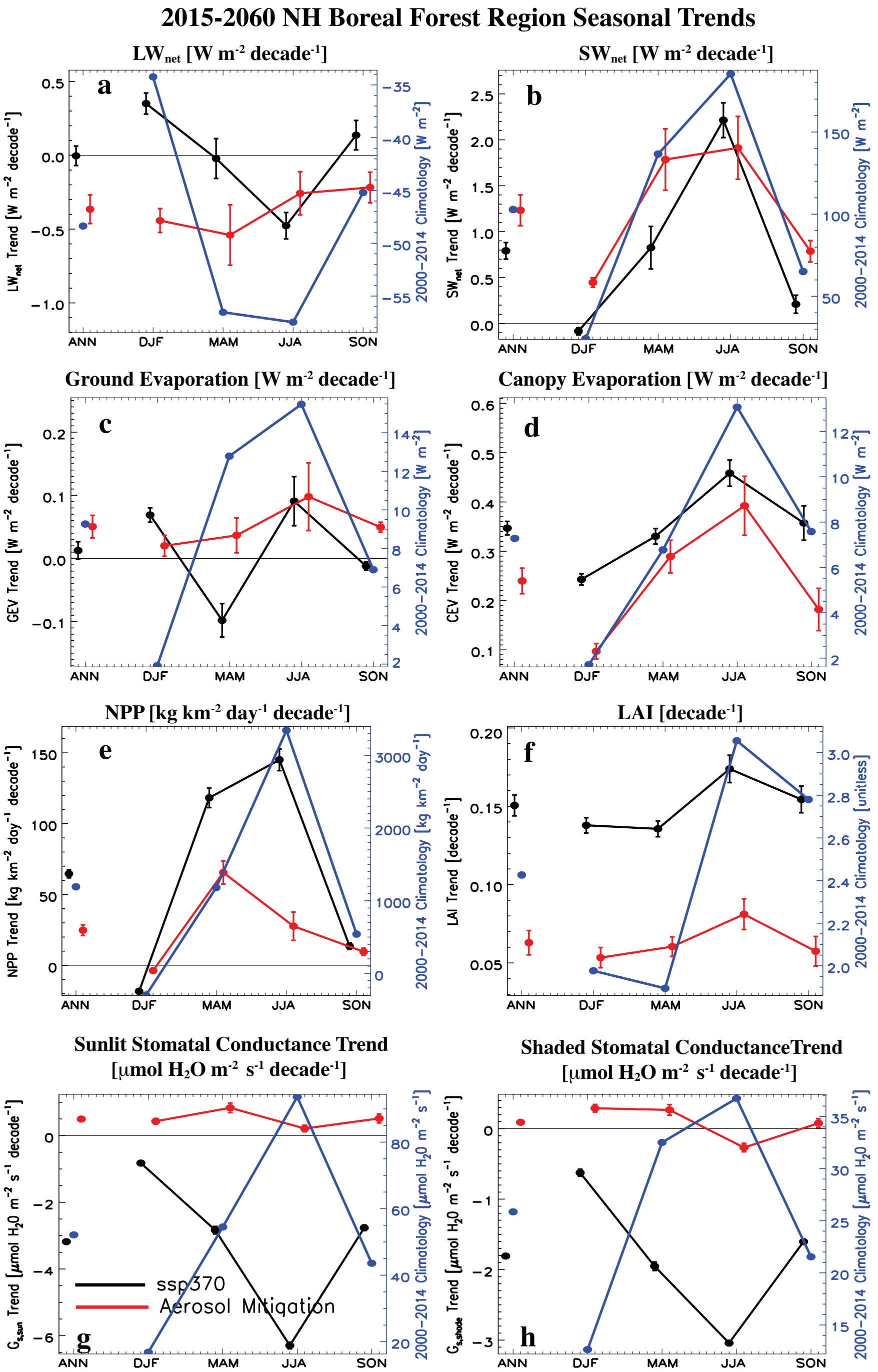

Supplementary Figure 12 **2015-2060 NH boreal forest region seasonal trends.** NH boreal forest region seasonal trends for (a) surface net longwave radiation (LW_{net}; [W m^{-2} decade^{-1}]); (b) surface net shortwave radiation (SW_{net}; [W m^{-2} decade^{-1}]; (c) ground evaporation [W m^{-2} decade^{-1}]; (d) canopy evaporation [W m^{-2} decade^{-1}]; (e) net primary productivity (NPP; [kg km^{-2} day^{-1} decade^{-1}]); (f) leaf area index (LAI; [decade^{-1}]); (g) sunlit stomatal conductance [μmol H_20 m^{-2} s^{-1} decade^{-1}]; and (h) shaded stomatal conductance [μmol H_20 m^{-2} s^{-1} decade^{-1}]. In addition to 2015-2060 ssp370 (black) and aerosol mitigation (red) trends, the 2000-2014 climatology (blue) is also included. Seasons include December-January-February (DJF), March-April-May (MAM), June-July-August (JJA) and September-October-November (SON). Also included is the annual mean (ANN). Error bars show the 90% confidence interval of the trend, estimated as $\frac{1.65\times\sigma}{\sqrt{n-1}}$, where σ is the standard deviation across the trends and n is the number of trends (i.e., 10).

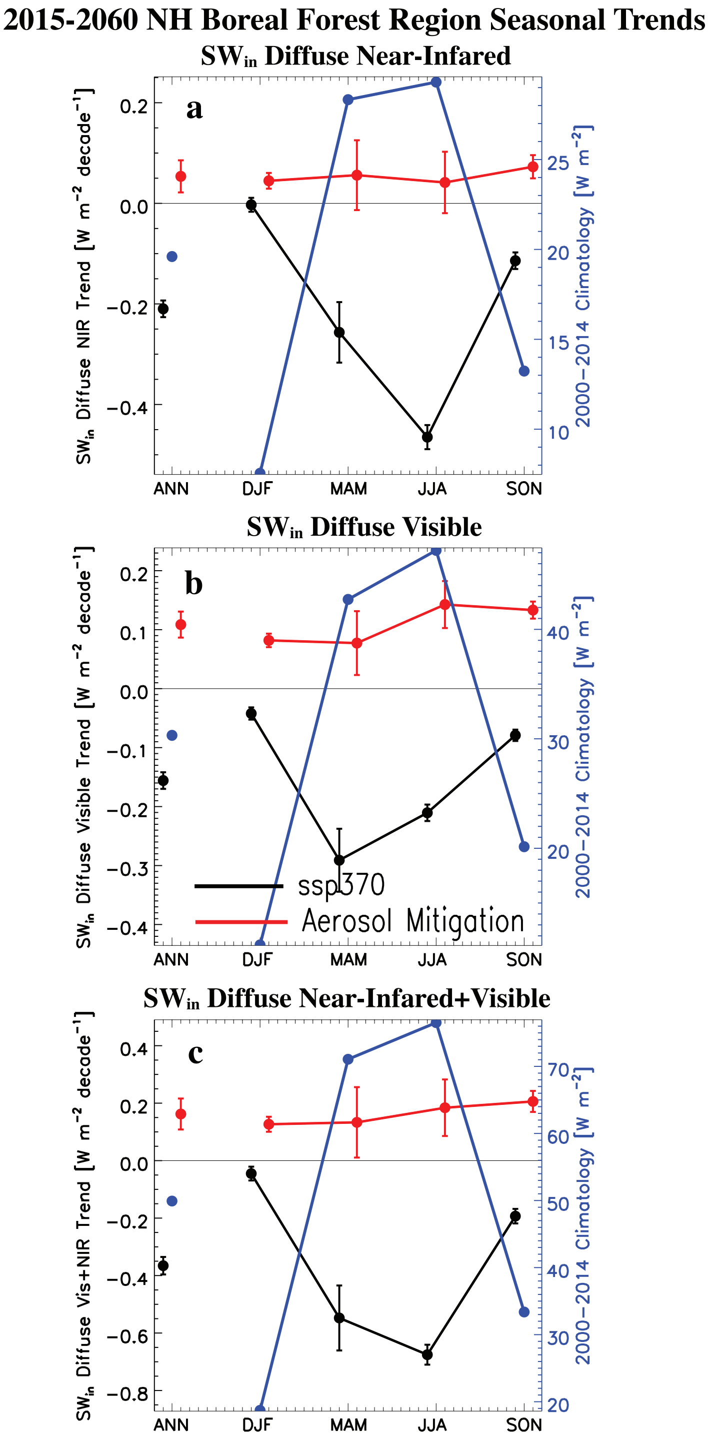

Supplementary Figure 13 **2015-2060 NH boreal forest region seasonal diffuse shortwave radiation trends.** NH boreal forest region seasonal trends for surface downwelling diffuse (a) near-infrared; (b) visible; and (c) visible+near-infrared shortwave radiation [W m^{-2} $decade^{-1}$] components. In addition to 2015-2060 ssp370 (black) and aerosol mitigation (red) trends, the 2000-2014 climatology (blue) is also included. Seasons include December-January-February (DJF), March-April-May (MAM), June-July-August (JJA) and September-October-November (SON). Also included is the annual mean (ANN). Error bars show the 90% confidence interval of the trend, estimated as $\frac{1.65\times\sigma}{\sqrt{n-1}}$, where σ is the standard deviation across the trends and n is the number of trends (i.e., 10).

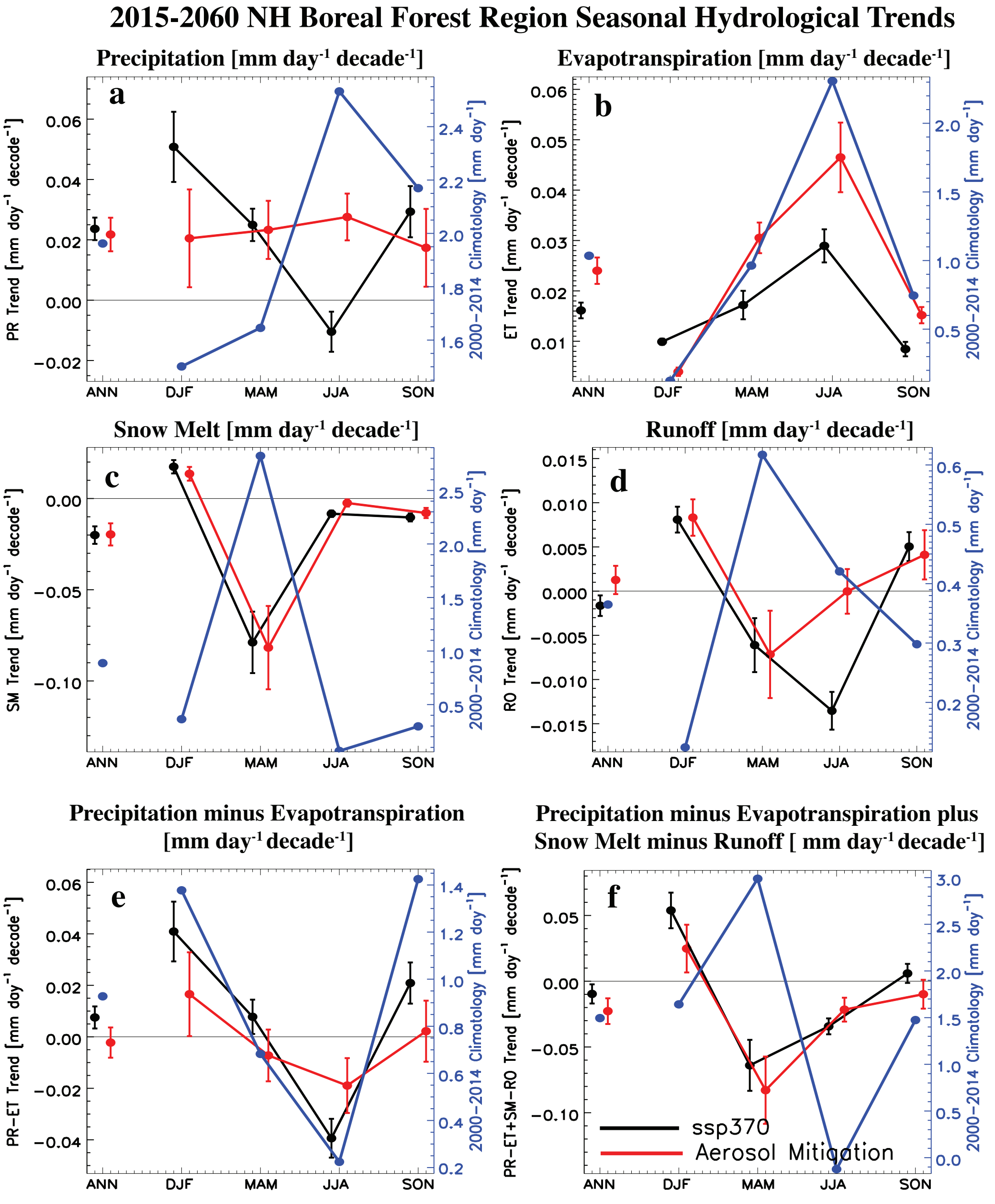

Supplementary Figure 14 **2015-2060 NH boreal forest region seasonal hydrological trends.** NH boreal forest region seasonal trends for (a) precipitation [mm day^{-1} decade^{-1}]; (b) evapotranspiration [mm day^{-1} decade^{-1}]; (c) snow melt [mm day^{-1} decade^{-1}]; (d) surface runoff [mm day^{-1} decade^{-1}]; (e) precipitation minus evapotranspiration [mm day^{-1} decade^{-1}] and; (f) precipitation minus evapotranspiration plus snow melt minus runoff [mm day^{-1} decade^{-1}]. In addition to 2015-2060 ssp370 (black) and aerosol mitigation (red) trends, the 2000-2014 climatology (blue) is also included. Seasons include December-January-February (DJF), March-April-May (MAM), June-July-August (JJA) and September-October-November (SON). Also included is the annual mean (ANN). Error bars show the 90% confidence interval of the trend, estimated as $\frac{1.65\times\sigma}{\sqrt{n-1}}$, where σ is the standard deviation across the trends and n is the number of trends (i.e., 10).

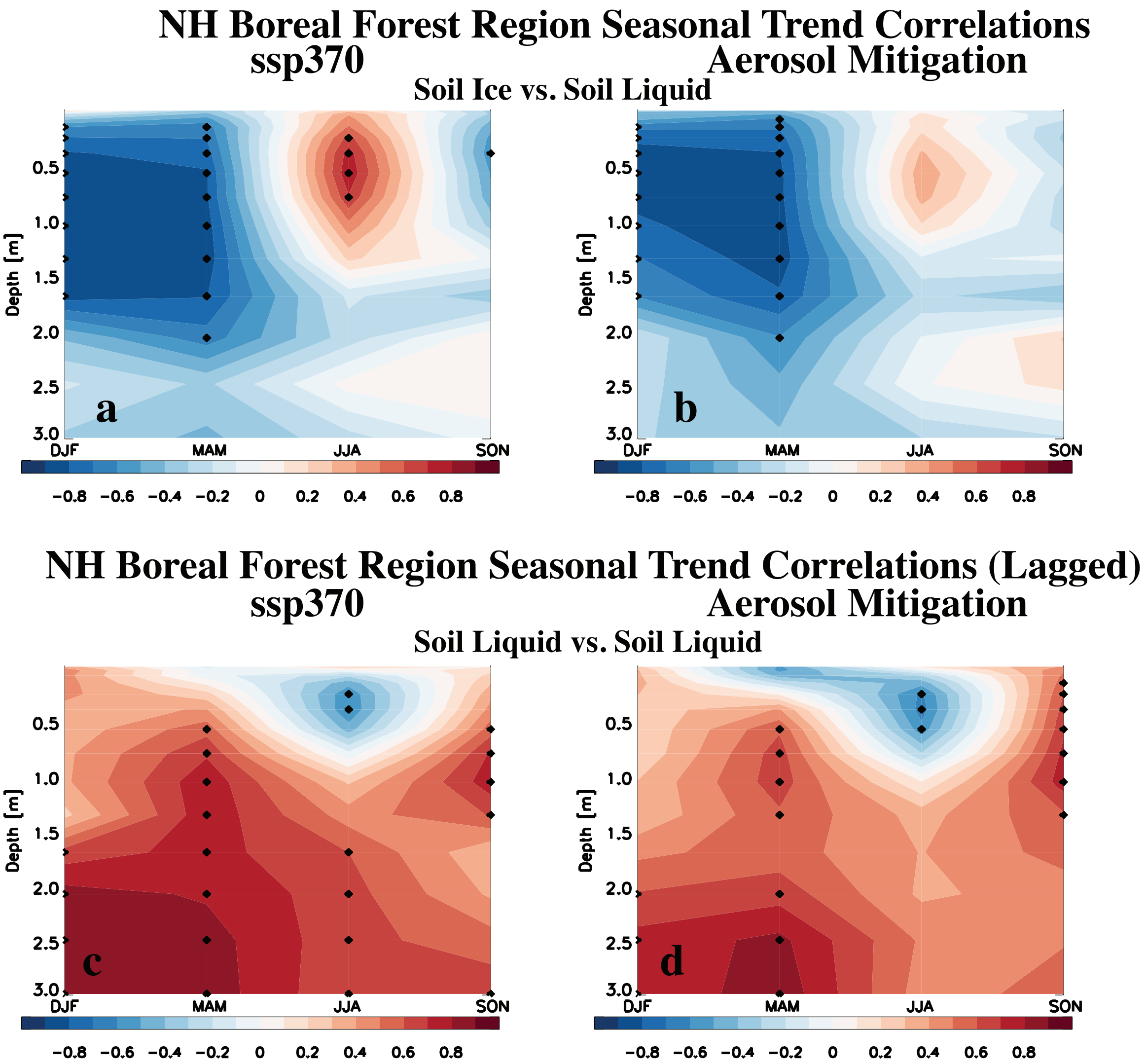

Supplementary Figure 15 **2015-2060 season versus depth soil trend correlations.** NH boreal forest region depth versus season soil liquid trend versus soil ice trend for (a) ssp370 and (b) aerosol mitigation. Bottom panels (c,d) show soil liquid versus soil liquid lagged trend correlations for (c) ssp370 and (d) aerosol mitigation. Seasons include December-January-February (DJF), March-April-May (MAM), June-July-August (JJA) and September-October-November (SON). Dots represent a significant correlation at the 90% confidence level based on a standard t-test. In panels (c,d), DJF shows the soil liquid trend correlation between DJF and the prior season, SON. Similarly, MAM shows the soil liquid trend correlation between MAM and the prior season, DJF, etc.

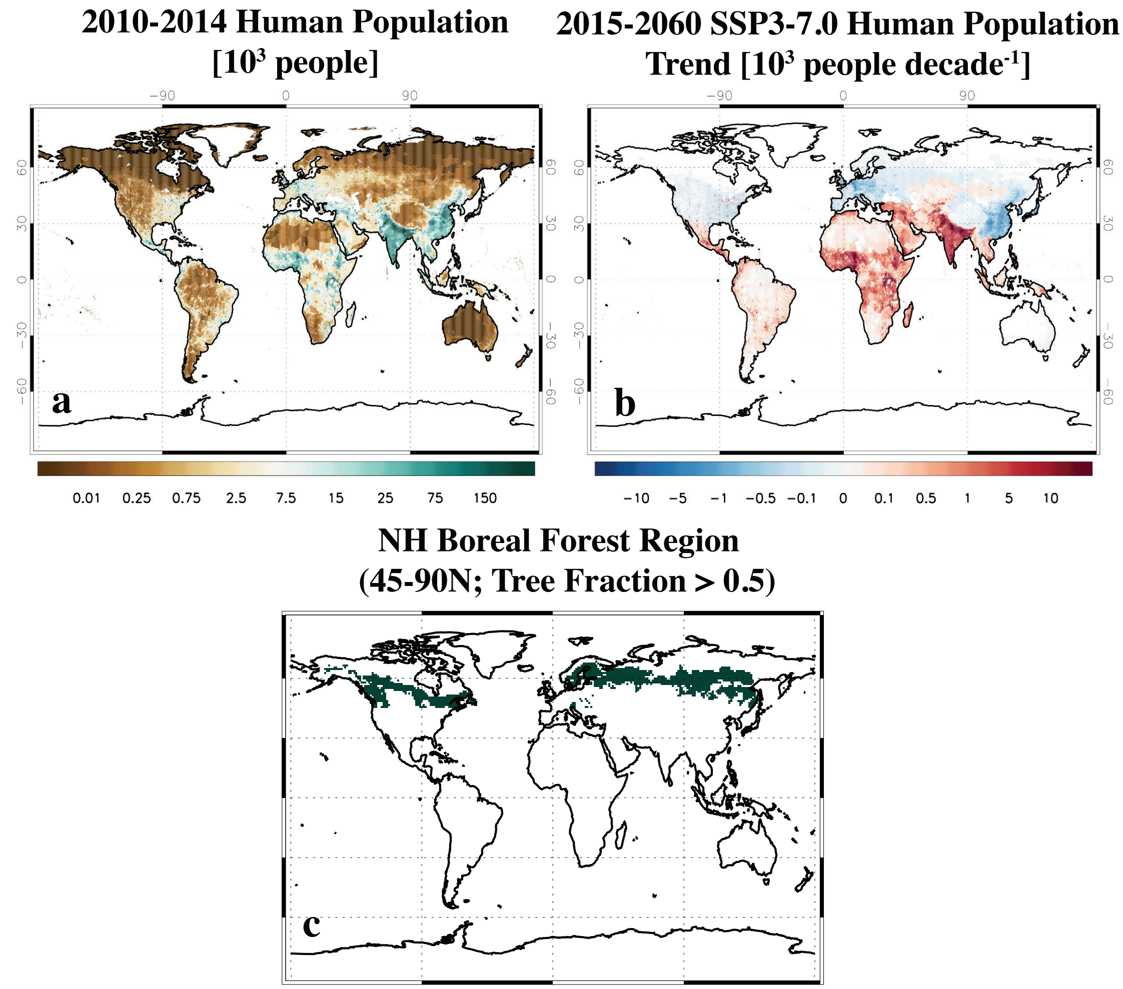

Supplementary Figure 16 **Human population climatology and trends.** Human population for the (a) 2010-2014 time period [10^3 people] and (b) 2015-2060 trends [10^3 people decade^{-1}] based on SSP3-7.0. Panel (c) shows the NH boreal forest region (i.e., dark green shading) as defined here, as grid boxes over land from 45-90°N with at least 50% tree fraction (as in Figure 1 from the main paper).

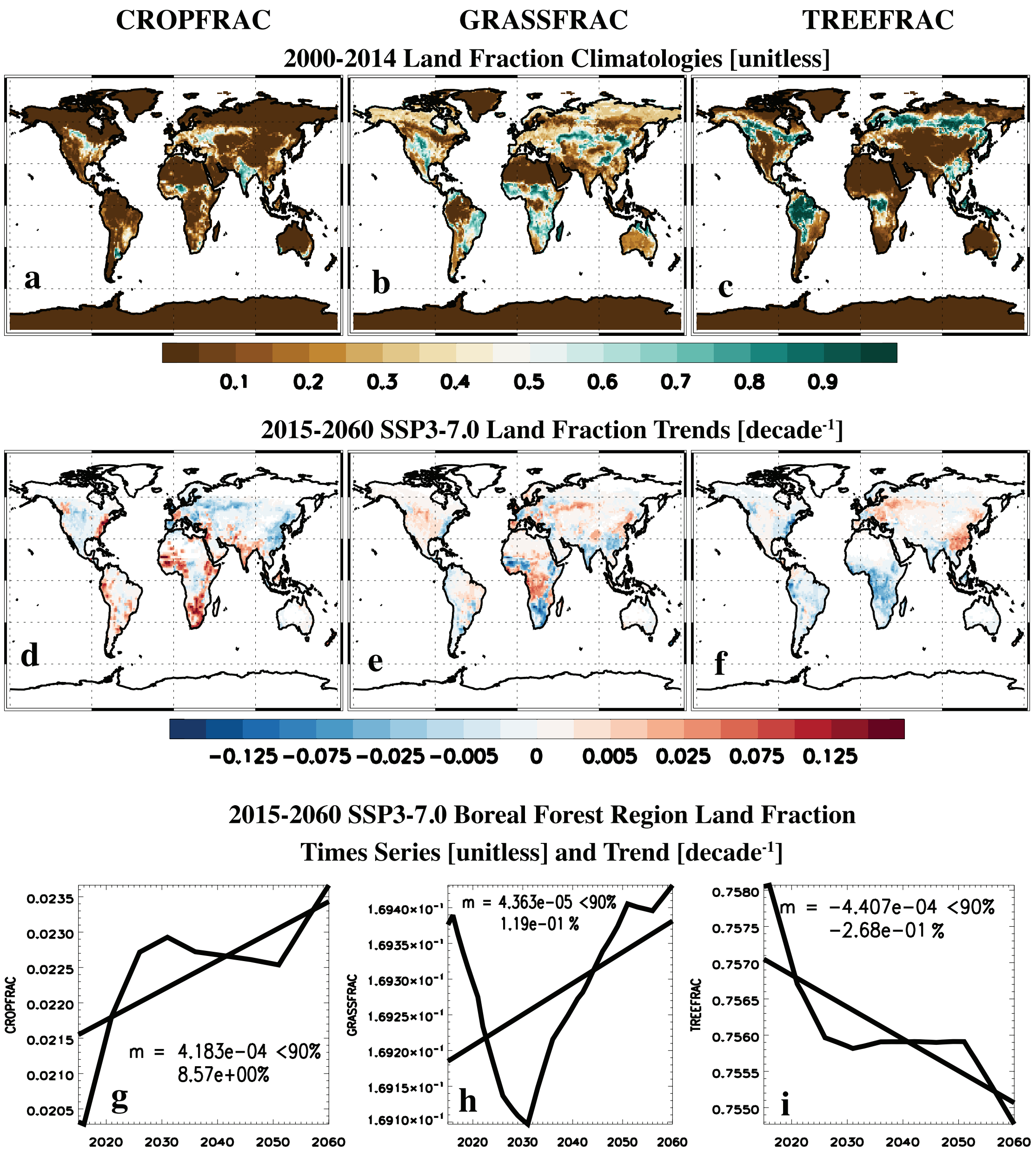

Supplementary Figure 17 **Land fraction climatology and trends.** Land fraction for (a, d, g) crops; (b, e, h) grasses; and (c, f, i) trees for the (a-c) 2010-2014 climatology [unitless]; (d-f) 2015-2060 trends [decade^{-1}] based on SSP3-7.0; and (g-i) NH boreal forest region 2015-2060 time series based on SSP3-7.0. In panels (g-i), we include the trend line, it's slope (denoted by "m") and significance level (e.g., all three are not significant at the 90% confidence level). Below this, we also include the percent change (estimated from the trend line).
